# Diagnosing soil bioremediation failure: evidence-weighted pathways from mechanism to field closure

**DOI:** 10.3389/fbioe.2026.1897329

**Published:** 2026-07-31

**Authors:** Agnieszka Mrozik, Zofia Piotrowska-Seget, Mariusz Cycoń

**Affiliations:** 1 Institute of Biology, Biotechnology and Environmental Protection, Faculty of Natural Sciences, University of Silesia, Katowice, Poland; 2 Department of Microbiology, Faculty of Pharmaceutical Sciences, Medical University of Silesia, Sosnowiec, Poland

**Keywords:** contaminant bioavailability and co-contamination, failure-mode diagnosis, omics and AI validation, PFAS immobilization and exposure control, residual-risk closure, soil bioremediation failure

## Abstract

Soil bioremediation is often presented as a sustainable alternative to physicochemical remediation, yet its field performance remains less predictable than laboratory and microcosm evidence suggests. This critical review argues that the central problem is not a lack of advanced technologies, but the frequent mismatch between the diagnosed failure mode, the selected intervention, and the endpoint used to claim success. Organic pollutants may be degraded, transformed, mineralized, or converted to hazardous intermediates, whereas metals and metalloids can only be immobilized, stabilized, extracted, accumulated, or shifted in speciation and bioavailability. Soils contaminated with per- and polyfluoroalkyl substances (PFAS)further expose the need to distinguish destruction from sorption, retention, leaching control, and exposure interruption. The review evaluates soil bioremediation through explicit criteria: mechanistic plausibility, soil realism, endpoint relevance, biological activity, temporal durability, field readiness, safety, scalability, and residual risk. The evidence indicates that underperformance commonly arises from mass-transfer limitation, contaminant aging, redox mismatch, oxygen and moisture heterogeneity, co-contamination, toxic intermediate accumulation, inoculant establishment failure, phytoremediation endpoint mismatch, and rebound after active treatment stops. Emerging tools are treated here as conditional evidence or conditional interventions, not as generic solutions. Nanomaterials are admissible as bioremediation only when they support a measured biological process rather than only sorption, catalysis, or immobilization. Omics and isotope-linked methods can strengthen causal inference, but closure still requires convergence with contaminant kinetics, intermediate control, toxicity reduction, and post-treatment stability. Synthetic microbial communities and engineered microbial systems must retain function after ecological filtering. Artificial intelligence and digital twins can structure decisions only within validated data domains and declared uncertainty bounds. The principal contribution of this review is a field-facing decision sequence that links failure mode, limiting mechanism, diagnostic evidence, admissible intervention, secondary-risk control, and closure. This framework shifts soil bioremediation from technology optimism toward diagnosis-led, evidence-weighted, risk-controlled deployment.

## 1 Introduction and operational definitions

Soil bioremediation occupies an unusual position in environmental science. Its biological premise is well established, but its field performance remains uneven. Microorganisms and plants can degrade, transform, immobilize, stabilize, accumulate, extract, or alter the bioavailability of contaminants. That breadth is scientifically useful, but it also creates a recurrent interpretive error: very different endpoints are often discussed as if they represented the same kind of success. Organic pollutants, including petroleum hydrocarbons, polycyclic aromatic hydrocarbons (PAHs), polychlorinated biphenyls (PCBs), pesticides, chlorinated solvents, dyes, and other xenobiotics, may undergo biodegradation, cometabolism, partial transformation, detoxification, or incomplete conversion to hazardous intermediates. Persistent fluorinated contaminants, particularly per- and polyfluoroalkyl substances (PFAS), require separate interpretation because apparent risk reduction may reflect sorption, retention, reduced pore-water concentration, or exposure interruption rather than biological destruction. PFAS-contaminated soils therefore sharpen the central evidentiary distinction of this review: closure must separate destruction, precursor transformation, immobilization, leaching control, and residual exposure (Liang et al., 2024; Chen H. et al., 2025). Metals and metalloids cannot be biologically degraded. Their risk can be reduced only through changes in speciation, oxidation state, solubility, sorption, precipitation, complexation, mobility, plant uptake, or exposure pathway. A critical evaluation must begin with these distinctions because they determine what evidence is admissible for remediation success (Vangronsveld et al., 2009; Sales da Silva et al., 2020; Praveen and Nagalakshmi, 2022; Mokrani et al., 2024; Hou et al., 2025).

The literature is strong in showing that biological systems can transform contaminants under defined conditions. It is weaker in explaining why those processes fail to deliver the expected result in soil. The difference is not trivial. A compound may persist because the degraders are absent, but also because it is aged, sorbed, trapped in a non-aqueous phase, separated from microbial cells by water discontinuity, exposed to the wrong redox regime, or coupled to a metal that suppresses enzyme activity. A phytoremediation treatment may fail not because the plant is unsuitable in a general sense, but because the intended endpoint was never defined: phytoextraction, phytostabilization, rhizodegradation, phytodegradation, and phytovolatilization carry different evidence burdens. A bioaugmentation trial may fail even when the introduced strain is competent in culture because field soil imposes competition, predation, phage pressure, dormancy, pore-scale dispersal limits, and loss of functional traits (Vogel, 1996; Semple et al., 2003; Diplock et al., 2009; Mrozik and Piotrowska-Seget, 2010; Gkorezis et al., 2016; Mawarda et al., 2020; Čaušević et al., 2024).

This mismatch between biological potential and field outcome explains why advanced tools should be evaluated as mechanism-bound instruments rather than as parallel remedies. Nanomaterials, omics, synthetic microbial communities (SynComs), and artificial intelligence (AI) can improve a remediation decision only when they address a diagnosed limitation, define a testable endpoint, and do not transfer risk to another compartment or to soil function. Their evidentiary value therefore depends on the failure mode, the validation level, and the residual risk introduced by the intervention (Sharma et al., 2022; Anifowose and Anifowose, 2024; Chen S. et al., 2024; Ruan et al., 2024).

**FIGURE 1:**
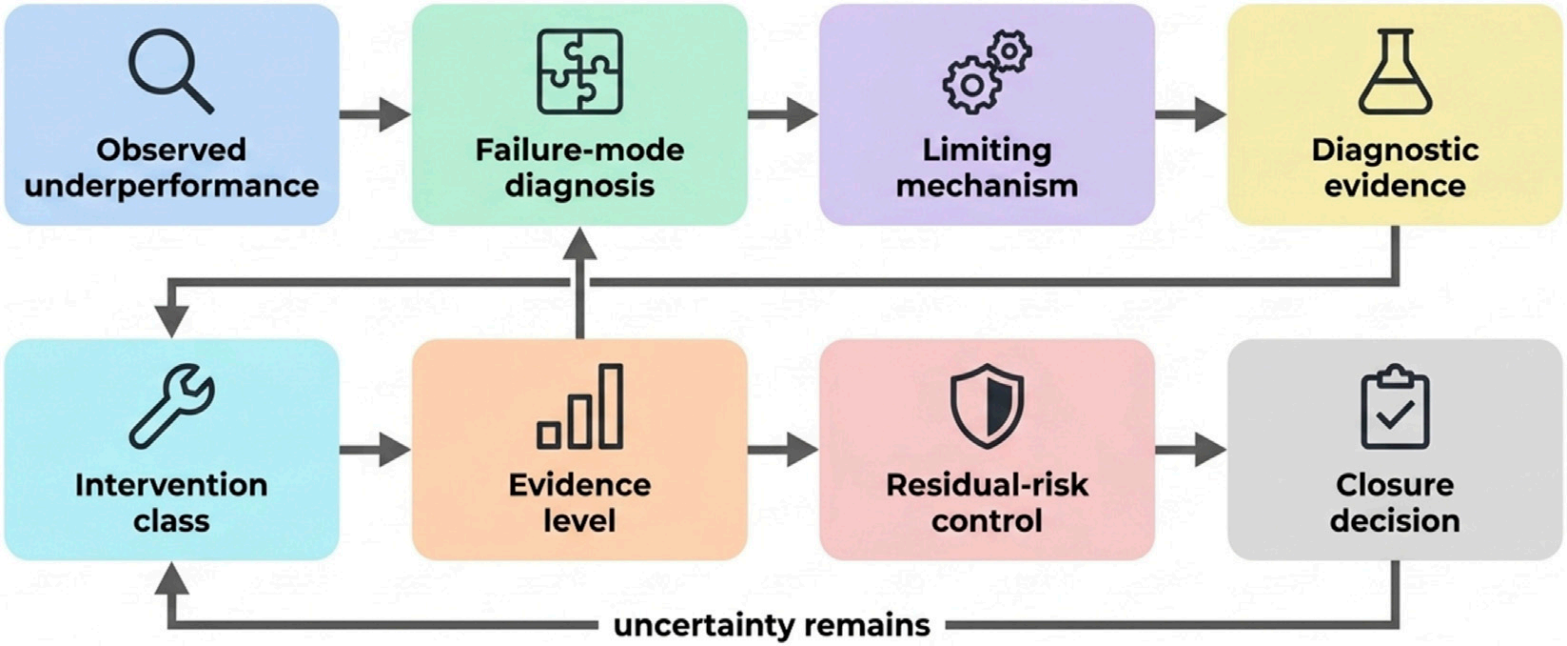
Main decision sequence for diagnosing and managing soil bioremediation failure. The framework treats underperformance as a diagnostic problem before intervention selection. The sequence moves from observed underperformance to failure-mode diagnosis, identification of the limiting mechanism, assessment of diagnostic evidence and evidence level, selection of an admissible intervention, residual-risk control, and closure decision. The figure shows decision order only; detailed diagnostic criteria and intervention conditions are provided in the tables.

The principal contribution of this review is a diagnostic decision grammar for soil bioremediation failure. Rather than organizing the evidence by technology class, the review asks six linked questions: what endpoint failed, which limiting mechanism explains the failure, what evidence supports that diagnosis, which intervention is admissible, what secondary risk is introduced, and what evidence is sufficient for closure. This structure separates degradation from transformation, immobilization, detoxification, exposure interruption, rebound control, and ecological recovery. Table 1 contrasts this approach with technology-centered review structures, and Figure 1 summarizes the resulting decision sequence.

**TABLE 1:** Technology-centered review focus versus the diagnostic contribution of the present review.

Review orientation	Field-decision gap	Diagnostic contribution of the present review
Technology overviews of microbial, plant-assisted, and metal (loid) remediation	Degradation, transformation, stabilization, extraction, immobilization, and exposure control are often discussed as comparable forms of success, although they support different closure claims	Separates contaminant classes and endpoints before evidence is interpreted; assigns different evidence requirements to organic transformation, metal (loid) risk reduction, PFAS retention, exposure control, and closure
Nano-remediation and nano-bioremediation reviews	Sorption, catalysis, immobilization, plant support, and biological compatibility can be merged under one technology label	Distinguishes physicochemical nanoremediation, nano-assisted bioremediation, and biologically mediated remediation; admits nano-enabled treatment as bioremediation only when a measured biological process contributes to the endpoint
Omics and molecular-monitoring reviews	Taxa, genes, transcripts, proteins, and metabolites can be overread as proof of pathway operation, detoxification, or closure	Ranks molecular evidence by inferential distance from verified activity; requires convergence with contaminant kinetics, transformation products, isotope or activity-linked evidence, toxicity, and durability for high-level claims
Bioaugmentation and SynCom reviews	Inoculant detection, designed membership, or short-term survival may be mistaken for retained function after ecological filtering in soil	Requires establishment, activity, endpoint change, safety assessment, and post-intervention monitoring before inoculation or SynComs can support decision claims
AI, machine-learning, and digital-twin reviews	Internal prediction accuracy may be treated as field evidence without a declared decision claim, uncertainty, validation design, or applicability domain	Defines AI and digital twins as decision-support tools; requires explicit decision use, independent validation where possible, uncertainty reporting, update rules, and applicability-domain control
Present review	Evidence organized by technology alone does not resolve whether underperformance has been correctly diagnosed or whether closure is justified	Links observed underperformance, failure mode, limiting mechanism, diagnostic evidence, admissible intervention, secondary-risk control, and closure in one decision sequence

AI, artificial intelligence; SynCom, synthetic microbial community. The table contrasts technology-centered review orientations with the diagnostic and decision-facing structure used in this review. The present review treats advanced tools as conditional responses to defined failure modes, not as parallel remedies.

This article is a critical review rather than a systematic review or meta-analysis. Its purpose is not to estimate pooled effect sizes, but to evaluate how different types of evidence support claims about failure mechanisms, intervention selection, field readiness, and closure. Literature searches were conducted in Web of Science, Scopus, PubMed, Google Scholar, ScienceDirect, and publisher platforms, including ACS Publications, SpringerLink, Wiley Online Library, MDPI, Elsevier, Frontiers, Springer Nature, and Taylor and Francis. The final search was completed on 30 April 2026, with update searches on 1 July 2026. The cited literature spans 1996–2026, with priority given to recent publications from 2022 to 2026. Earlier foundational studies were retained when they introduced a mechanism, diagnostic concept, field precedent, isotope method, or regulatory endpoint that remains directly relevant to soil bioremediation failure. Searches were restricted to English-language publications and relevant institutional guidance documents. Search strings combined contaminant, process, evidence, and decision terms, including soil bioremediation, phytoremediation, bioaugmentation, biostimulation, nano-enabled remediation, nanoremediation, omics, metagenomics, metatranscriptomics, metaproteomics, metabolomics, stable-isotope probing, compound-specific isotope analysis, SynComs, engineered microorganisms, clustered regularly interspaced short palindromic repeats (CRISPR), artificial intelligence, digital twin, field validation, monitoring, residual risk, closure, rebound, bioavailability, redox limitation, co-contamination, toxic intermediates, PFAS, petroleum hydrocarbons, PAHs, PCBs, pesticides, chlorinated solvents, metals, and metalloids. Studies were included when they addressed at least one of the following: a mechanistic cause of reduced remediation performance, a diagnostic method linked to contaminant fate or biological activity, an intervention tested in soil or soil-relevant systems, field or pilot-scale performance, safety constraints, monitoring, residual risk, or decision support for remediation. Studies were excluded from evidentiary claims when they were limited to generic technology promotion, non-soil systems without clear transferability, contaminant disappearance without endpoint definition, or prediction without validation. The review follows SANRA principles for critical narrative quality and selected PRISMA 2020 reporting elements for transparency, without claiming PRISMA compliance (Baethge et al., 2019; Page et al., 2021; Gonot-Schoupinsky et al., 2022). The evidence-weighting rubric in Table 2 was applied qualitatively rather than as an additive score. Field, pilot-scale, field-cell, or near-operational studies in historically contaminated, non-sterile, well-characterized soil were given greater decision weight than culture, sterile-soil, freshly spiked, or short-incubation studies. For closure claims, endpoint definition, temporal depth, spatial design, post-treatment durability, and secondary-risk assessment were weighted more strongly than removal efficiency. For biological-attribution claims, activity-linked evidence, including transcripts, proteins, metabolites, stable isotope probing (SIP), isotope evidence, enzyme assays, functional assays, or strain-specific activity tracking, was weighted more strongly than taxa, total DNA, or inoculant persistence alone. Studies lacking endpoint definition, validation, or secondary-risk assessment were used only as mechanistic or contextual evidence unless they provided a unique observation unavailable from stronger evidence.

**TABLE 2:** Evidence-weighting rubric used in this critical review.

Evidence feature	Higher evidentiary weight	Lower evidentiary weight	Interpretive consequence
Study setting	Field, pilot, field cell, or near-operational soil study	Culture, simplified matrix, sterile soil, short incubation	Field claims require field-relevant evidence
Soil realism	Historically contaminated, non-sterile, characterized soil	Freshly spiked, homogenized, or poorly characterized soil	Real-soil evidence carries greater diagnostic value
Endpoint definition	Parent compound plus bioavailable fraction, intermediates, toxicity, leachability, or metal speciation	Parent-compound decrease or total concentration alone	Removal is not equivalent to detoxification or closure
Biological inference	Activity-linked evidence, such as transcripts, proteins, metabolites, SIP, enzyme assays, or functional assays	Taxonomic shifts, total DNA, or culture phenotype alone	Presence or potential should not be treated as activity
Temporal depth	Repeated monitoring during and after intervention	Single endpoint or sampling only during treatment	Durability requires post-intervention evidence
Spatial design	Sampling resolves heterogeneity, depth, and treatment distribution	Sparse, pooled, or unreported spatial design	Poor spatial design weakens closure relevance
Intervention traceability	Dose, delivery, material properties, inoculum identity, carrier, or model inputs reported	Poorly specified treatment or black-box prediction	Non-traceable interventions cannot support reproducibility
Safety and secondary risk	Toxicity, leaching, biomass fate, nanoparticle fate, ecological displacement, or rebound assessed	Efficiency reported without secondary-risk assessment	Risk transfer limits field readiness
Model validation	Independent validation, uncertainty, metrics, and applicability domain reported	Calibration-only model or prediction without decision claim	AI evidence is decision support, not field proof

AI, artificial intelligence; SIP, stable isotope probing. The rubric was used as a qualitative evidence-weighting guide, not as an additive scoring system. Evidence was treated as decision-relevant when the features most material to the claim were present. For closure claims, field setting, endpoint definition, temporal depth, spatial design, and secondary-risk assessment were weighted more strongly than laboratory efficiency. For biological-attribution claims, activity-linked evidence was weighted more strongly than taxonomic shifts or DNA-based potential alone. Studies lacking endpoint definition, validation, or secondary-risk assessment were not used to support field-readiness or closure claims, even when they contributed mechanistic or contextual evidence.

Five terms are used operationally throughout this review. Failure means that the selected biological or plant-assisted intervention does not produce the endpoint required for the contaminant class, exposure pathway, and intended land use. Underperformance means a weaker condition: measurable improvement occurs, but the rate, extent, durability, toxicity reduction, or exposure control remains insufficient for the declared remediation objective. Field readiness means that a process has been demonstrated in non-sterile, physically structured, chemically characterized soil at a scale and duration relevant to delivery, monitoring, safety, and post-treatment durability. Decision-grade evidence means evidence strong enough to justify a remediation decision, not merely to support mechanistic plausibility; it requires alignment among contaminant chemistry, biological activity where claimed, spatial and temporal design, secondary-risk assessment, and uncertainty. Closure means a risk-based endpoint at which additional active treatment is not justified because residual contaminant mass, bioavailability, toxicity, leaching, vapor or plant exposure, rebound potential, and intended land use have been evaluated together.

## 2 Failure-mode diagnosis in soil bioremediation

The weakest form of interpretation in soil bioremediation is to treat poor contaminant removal as a single outcome with a single cause. In real soil, the same endpoint can have different origins. Slow PAH removal may reflect missing degraders, but it may also reflect contaminant aging, sorption, diffusion limitation, or low pore-water concentration. Incomplete chlorinated solvent remediation may reflect insufficient donor, unsuitable redox potential, competition from sulfate reduction or methanogenesis, or absence of a microbial guild capable of complete dechlorination. Low metal risk reduction may reflect poor amendment choice, but also pH change, dissolved organic matter, competing ions, root activity, or redox-driven remobilization. A critical evaluation must therefore separate symptoms from mechanisms. The strongest evidence across the field supports three broad diagnostic starting points. These classes are not mutually exclusive categories; final attribution requires testing the dominant limiting process. The first is physicochemical inaccessibility, where contaminants and organisms are present but poorly connected. This includes sorption to organic matter or minerals, non-aqueous phase liquid (NAPL) entrapment, contaminant aging, water discontinuity, diffusion limitation, and low bioavailable fraction. The second is geochemical misalignment, where the dominant redox state, electron donor supply, electron acceptor supply, pH, moisture, or oxygen distribution does not match the pathway required for the contaminant. The third is biological or endpoint mismatch, where the desired function is absent, inactive, ecologically unstable, or measured through the wrong endpoint. Because these processes commonly co-limit field performance, attribution should identify the dominant limitation and the co-limitation most likely to invalidate the selected intervention. The diagnostic sequence is important because these failure classes can mask one another. A soil may appear biologically inactive because the relevant organisms are missing, but the same observation can arise when the contaminant is present only in a slowly desorbing pool or when oxygen and water films do not intersect the contaminated domain. Conversely, a highly active microbiome may coexist with poor remediation if the measured endpoint is total concentration rather than the fraction that drives exposure. Diagnosis must therefore begin with the limiting process, not with the preferred intervention.

Bioavailability is the clearest example of an established mechanism that is still often overinterpreted. It is well supported that aged hydrophobic contaminants can become less accessible to microorganisms through sorption, sequestration, and slow desorption (Alexander, 2000; Semple et al., 2003; Kanaly and Harayama, 2010). What remains less often resolved is whether an intervention reduces risk or merely changes extractability. Increasing contaminant availability may improve biodegradation when microbial capacity and electron acceptors are sufficient. It may also increase leaching or exposure when degradation does not keep pace. For this reason, bioavailability interventions should be evaluated by pore-water concentration, desorption behavior, toxicity, and leaching, not by total concentration alone. Additional mass-transfer studies strengthen this interpretation when they distinguish diffusion, desorption and pore-scale access from biological capacity (Bosma et al., 1997; García-Rivero et al., 2002). Soil-matrix studies that pair accessibility with microbial response carry greater interpretive value, but they remain conditional when they stop at disappearance from an extract without toxicity, leaching or closure endpoints (Lyu et al., 2014; Haghollahi et al., 2016; Haleyur et al., 2019; Martínez Álvarez et al., 2020).

Redox limitation is equally important, but its interpretation depends on contaminant class. Aerobic hydrocarbon degradation and reductive dechlorination require different electron-flow architectures. Adding oxygen can assist many hydrocarbon pathways but can suppress reductive dechlorination. Adding donor can promote organohalide respiration, but excessive donor can be consumed by sulfate reducers, iron reducers, or methanogens rather than by the intended pathway (Fennell et al., 1997; Amellal et al., 2001; Meckenstock et al., 2015; Sabeti Mohammadi et al., 2021; Davis, 2023). The unresolved issue is not whether redox conditions matter. It is whether studies diagnose the dominant terminal electron-accepting process before interpreting success or failure. Evidence on climate-sensitive microbial activity supports the same diagnostic caution because temperature and moisture can change pathway rates without changing the apparent contaminant class (Alkorta et al., 2017). Evidence on acidification and metal-linked functional shifts adds a second boundary condition, because pH and metal stress can suppress microbial functions even when organic substrates and degraders are present (Anza et al., 2021). Treatment performance should therefore be interpreted against soil boundary conditions, not contaminant concentration alone.

**FIGURE 2:**
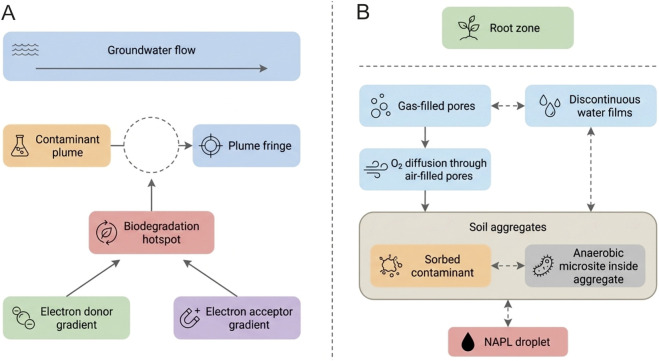
Saturated plume logic **(A)** versus vadose-zone soil logic **(B)** in contaminant biodegradation. The figure contrasts a saturated plume-fringe model with the discontinuous transport structure of unsaturated soil. In the vadose zone, water films, gas-filled pores, sorption domains, NAPL entrapment, and aggregate microsites can separate microorganisms from contaminants and electron acceptors. Arrows show transport or gradient direction, not confirmed biodegradation. NAPL, non-aqueous phase liquid.

The vadose zone adds another layer of difficulty. The plume-fringe concept can be useful for saturated groundwater, where advective transport and mixing define contact between electron donors and acceptors. Unsaturated soil behaves differently. Gas-filled pores, discontinuous water films, aggregate microsites, fluctuating moisture, and sorbed or trapped contaminant domains can separate microorganisms from substrates and electron acceptors even when bulk measurements appear favorable (Diplock et al., 2009; Brusseau et al., 2013; Shi et al., 2024). Field interpretation should therefore avoid assigning a single redox or oxygen status to soil without considering depth, pore structure, water content, and microscale heterogeneity. This distinction is summarized in Figure 2, which separates saturated plume-fringe logic from the discontinuous transport and contact architecture of vadose-zone soil.

Co-contamination is another case where the literature shows a consistent problem but still lacks sufficient decision rules. Many contaminated soils contain organic pollutants and metals together. Metals can inhibit microbial respiration, enzyme activity, membrane integrity, and degrader abundance, while organic pollutants and amendments can change metal mobility. Treating organic degradation and metal stabilization as independent tasks is therefore scientifically weak (Olaniran et al., 2013; Ali et al., 2022; Gil-Díaz et al., 2022; Pacwa-Płociniczak et al., 2024). In mixed pollution, the first decision should be endpoint hierarchy: degrade organics first, immobilize metals first, extract metals, reduce toxicity, or interrupt exposure. Without that hierarchy, integrated remediation becomes a slogan rather than a strategy.

A further failure arises when transformation is confused with detoxification. Parent-compound decline can be desirable, but only if hazardous intermediates are absent, transient, or further transformed. Chlorinated ethene remediation illustrates the principle: tetrachloroethene (PCE) and trichloroethene (TCE) decline is not sufficient if dichloroethene (DCE) isomers or vinyl chloride accumulate. Similar caution applies to chlorophenols, PCBs, PAHs, and pesticides when partial transformation produces reactive or persistent products (Bursian et al., 2010; Schroeder et al., 2012; Yadid et al., 2013; Fuentes et al., 2014; Sabeti Mohammadi et al., 2021). The minimum evidence standard for such pathways must include parent compounds, expected intermediates, redox status, biological activity where claimed, and toxicity or risk-relevant endpoints. This point is especially important for closure-oriented interpretation. A pathway that reduces an analytical parent compound but increases toxicity, mobility or exposure has not solved the remediation problem. The stronger evidentiary claim is pathway completion or risk reduction; partial transformation is only an intermediate observation unless the products and biological harm are resolved. The minimum analytical set should therefore be defined by contaminant class and pathway, not by a generic removal metric. Table 3 defines the minimum analyte and endpoint set needed to support remediation claims for selected contaminant classes. It is intended to prevent closure or detoxification claims based only on parent-compound decline, total concentration, sorption, or apparent removal without pathway, toxicity, mobility, or exposure evidence.

**TABLE 3:** Minimum analytical evidence required for risk-relevant claims across major contaminant classes and pathways.

Contaminant class or pathway	Minimum analyte and endpoint set	Claim that should be rejected without these data
PCE/TCE reductive dechlorination	PCE, TCE, *cis*-DCE, *trans*-DCE, 1,1-DCE where relevant, vinyl chloride, ethene, ethane, chloride release where feasible, donor and electron-acceptor trends, pH, Eh, methane, sulfide, and functional markers such as *tceA*, *vcrA*, *bvcA*, and *Dehalococcoides* abundance when biological attribution is claimed	Detoxification or closure based only on PCE/TCE decline
PAHs and petroleum hydrocarbons	Parent PAHs, alkylated PAHs where petroleum-derived, oxygenated or nitrated PAHs where expected, bioavailable or pore-water fraction, desorption behavior, toxicity bioassay, and mineralization or product control where degradation is claimed	Risk reduction based only on solvent-extractable parent decline
PCBs and chlorophenols	Congener or homolog profile, expected hydroxylated or chlorinated products, toxicity, and pathway markers where microbial transformation is claimed	Detoxification based only on total PCB or parent chlorophenol decline
Pesticides	Parent compound, known regulated metabolites, transformation products predicted from pathway evidence or suspect screening, leaching, and bioassay where toxicity is plausible	Successful remediation based only on disappearance of the parent pesticide
Metals and metalloids	Total concentration for inventory, pore-water or bioaccessible fraction, speciation or sequential extraction, pH, Eh, leaching, plant tissue burden where vegetation is present, and stability under likely site changes	Metal remediation based on total concentration alone
PFAS	Target PFAS including chain-length distribution, pore-water and leachate PFAS, precursor assessment where relevant, extractable organic fluorine or total oxidizable precursor assay where available, sorbent stability, and mass balance when destruction is claimed	Destruction or detoxification based only on sorption, lower pore-water concentration, or lower leaching

*bvcA*, vinyl chloride reductase gene associated with vinyl chloride-to-ethene dechlorination; *cis*-DCE, *cis*-1,2-dichloroethene; 1,1-DCE, 1,1-dichloroethene; Eh, redox potential; PAH, polycyclic aromatic hydrocarbon; PCB, polychlorinated biphenyl; PCE, tetrachloroethene; PFAS, per- and polyfluoroalkyl substances; TCE, trichloroethene; *tceA*, trichloroethene reductive dehalogenase gene; *trans*-DCE, *trans*-1,2-dichloroethene; *vcrA*, vinyl chloride reductive dehalogenase gene.

The biological side of failure is often described too narrowly as lack of degraders. The stronger interpretation is ecological establishment failure. In bioaugmentation and microbiome engineering, the first barrier is not only pathway competence, but establishment in the receiving soil. Introduced cells must survive release, reach the contaminated domain, compete or cooperate with resident taxa, avoid predation and phage-mediated losses, retain functional traits, and remain metabolically active under site-specific geochemistry. Detecting an inoculant is therefore not proof of function. Function requires evidence of activity and endpoint change (Vogel, 1996; Mrozik and Piotrowska-Seget, 2010; Mawarda et al., 2020; Albright et al., 2022; Čaušević et al., 2024; Papin et al., 2024). The same principle applies to SynComs. Designed composition at application is not field evidence unless function persists after ecological filtering. Earlier bioaugmentation and restoration studies are useful for identifying ecological constraints, but they are not decision-grade when strain selection or inoculation success is the primary endpoint (Thompson et al., 2005; Radwan et al., 2019). More recent inoculation studies are weighted more strongly when they connect establishment with activity, contaminant endpoints and soil-function recovery; otherwise they remain conditional evidence even when they report improved performance (Dadzie et al., 2024; Gangola et al., 2024). The minimum evidence for bioaugmentation should therefore include monitoring after dosing or active stimulation has stopped. Field evidence is strongest when inoculant or consortium tracking is paired with functional-gene monitoring, parent-daughter chemistry, ethene formation where relevant, and a post-intervention window capable of detecting rebound or loss of dechlorinating activity (Krett et al., 2024; Cycoń, 2026a).

Phytoremediation brings a final interpretive tension. Plant survival, contaminant uptake, reduced translocation, rhizosphere degradation, and soil stabilization cannot be treated as the same endpoint. Phytoextraction requires transfer to harvestable biomass and safe biomass handling. Phytostabilization requires reduced mobility or exposure. Rhizodegradation requires transformation in the root zone. Phytovolatilization requires assessment of atmospheric transfer. A treatment that increases uptake may be successful for extraction and unacceptable for stabilization. A treatment that decreases shoot transfer may be successful for stabilization and irrelevant for extraction (Vangronsveld et al., 2009; Gkorezis et al., 2016; Willscher et al., 2017; Shen et al., 2022; Pacwa-Płociniczak et al., 2023). Plant-assisted evidence is weighted first by endpoint specificity. Studies of microbial inoculation, mercury-resistant plant-growth-promoting rhizobacteria and pH-dependent plant performance support phytoremediation only when the plant response is tied to contaminant fate or exposure control (Akhtar et al., 2020; González et al., 2021; Razmi et al., 2021). Evidence from rhizosphere change and polymetallic systems is interpreted more cautiously, because improved vegetation, altered microbial communities or increased tissue accumulation can support different conclusions unless biomass fate, metal mobility and exposure are resolved (Durante-Yánez et al., 2022; Saldarriaga et al., 2023; Wu et al., 2024). For phytoremediation, the minimum evidence must also include the fate of contaminated biomass, the exposure pathway that the plant system is meant to interrupt, and the stability of the effect after the relevant growing season. A plant-based treatment should not be closed on the basis of growth, uptake, or reduced soil concentration alone when senescence, harvest, trophic transfer, leaching, or metal remobilization remains unresolved.

The major diagnostic categories discussed above are condensed in Table 4. The table is intended as a decision aid, not as a substitute for site-specific conceptual modeling; it links each failure mode to the evidence needed to avoid the most common overinterpretation. The literature supports a firm conclusion: bioremediation failure should not be assigned to generic “unfavorable conditions” or solved by default technological escalation. The first task is to identify the limiting mechanism. Some failures require geochemical correction, some require availability management, some require endpoint redefinition, some require pathway completion, and some require biological establishment evidence. This distinction sets the standard for the next section. Nano-enabled remediation should be judged only by whether it corrects one of these diagnosed mechanisms without creating a larger residual risk.

**TABLE 4:** Diagnostic interpretation of major soil bioremediation failure modes.

Failure mode	Diagnostic evidence	Common co-limitation	Common overinterpretation	Intervention implication
Low bioavailability or mass transfer	Desorption kinetics, pore-water concentration, passive sampling, toxicity	Redox heterogeneity, low moisture, insufficient degrader activity	Low removal means lack of degraders	Control availability safely
Oxygen limitation	Depth-resolved oxygen, moisture, respiration, redox microsites	Water discontinuity, organic-matter demand, soil compaction	Bulk oxygen reflects pore-scale oxygen availability	Restore aeration and moisture
Reductive pathway stall	Parent-daughter sequence, ethene, donor and acceptor trends, redox status	Donor competition, sulfate reduction, methanogenesis, missing dechlorinating guild	Parent loss equals detoxification	Rebalance donor and redox
Donor competition	Sulfide, methane, Fe(II), nitrate or sulfate depletion	Incomplete pathway, electron donor over-supply	More donor always improves degradation	Avoid donor oversupply
Co-contamination toxicity	Metal speciation, enzyme activity, toxicity, catabolic markers	pH shift, dissolved organic matter, organic amendment effects on metal mobility	Mixed pollution can be treated as independent single pollutants	Set endpoint hierarchy
Toxic intermediate accumulation	Daughter products, metabolites, toxicity, pathway markers	Redox mismatch, missing downstream metabolism	Parent-compound decline means risk reduction	Verify detoxification
Inoculant establishment failure	Strain-specific qPCR or ddPCR, transcripts, SIP, functional assays	Low bioavailability, predation, phages, dispersal limits, geochemical stress	Inoculation or survival means activity	Verify functional establishment
Phytoremediation endpoint mismatch	Tissue burden, translocation, leachability, rhizosphere activity, biomass fate	Metal remobilization, plant stress, biomass exposure	Increased uptake and decreased uptake are both “success”	Specify plant endpoint
Metal remobilization	Pore-water metal, sequential extraction, pH, Eh, leaching	Root-zone chemistry, wetting-drying, organic amendments	One-time immobilization means durable risk reduction	Stress-test stability
PFAS retention without destruction	Pore-water PFAS, leachate PFAS, precursor indicators, sorbent stability	Short-chain PFAS mobility, aging of sorbent, co-contaminant competition	Lower pore-water concentration means destruction	Do not claim destruction

ddPCR, droplet digital polymerase chain reaction; Eh, redox potential; PFAS, per- and polyfluoroalkyl substances; qPCR, quantitative polymerase chain reaction; SIP, stable isotope probing.

### 2.1 Field and pilot-scale illustrations of failure-mode diagnosis

Documented field and pilot-scale studies (Table 5) clarify how the proposed framework should be used. They are not presented as proof that a technology class is generally field-ready. Their value is diagnostic: each example links a limiting mechanism, a corrective intervention, and the endpoint that can or cannot support closure.

**TABLE 5:** Field and pilot-scale examples illustrating diagnostic interpretation of soil bioremediation and integrated remediation.

Study and scale	Diagnosed limitation	Corrective intervention	Outcome and interpretation	References
As- and Hg-contaminated brownfield soil, pilot field plots	Metal (loid) mobility and exposure risk, not degradable contaminant mass	Commercial nZVI amendment	The endpoint was immobilization and stability of As and Hg under field conditions, with 32-month monitoring. The evidence supports metal (loid) risk reduction, not bioremediation or contaminant destruction	Gil-Diaz et al. (2019)
Cr-rich technosol, field reclamation with *Capsicum annuum*	Plant establishment and Cr exposure in contaminated technosol	Compost plus arbuscular mycorrhizal fungi and *Aspergillus terreus*	Improved plant performance and reduced substrate Cr can support phytostabilization or exposure-control claims only if plant tissue burden, edible-tissue exclusion, biomass fate, and metal mobility are resolved	Akhtar et al. (2020)
Pilot-scale petroleum hydrocarbon phytoremediation	Hydrocarbon removal under plant-assisted field-relevant conditions	*Paspalum scrobiculatum* with process optimization	The study is useful as pilot-scale performance evidence, but optimization metrics do not replace contaminant-product chemistry, toxicity, moisture, delivery, and post-treatment persistence	Sanusi et al. (2016)
HCH-contaminated soil assessed with CSIA	Distinguishing biodegradation from concentration change caused by non-destructive processes	Nutrient amendment and bioaugmentation with an HCH-degrading bacterium	Isotope fractionation strengthened attribution of biodegradation, while the closure claim still required chemical endpoints and post-treatment risk interpretation	Qian et al. (2019)

CSIA, compound-specific isotope analysis; HCH, hexachlorocyclohexane; nZVI, nano zero-valent iron.

## 3 Nano-enabled soil remediation: mechanism-specific benefit under unresolved fate and toxicity constraints

Nano-enabled remediation should be separated into three evidence classes. The first is physicochemical nanoremediation, in which the primary process is sorption, complexation, precipitation, redox reaction, catalysis, photocatalysis, or contaminant retention by the material. This class may reduce mobility, pore-water concentration, or exposure, but it is not bioremediation unless a biological process is also measured. The second is nano-assisted bioremediation, in which a nanomaterial modifies a limiting condition for microbial or plant activity, for example electron transfer, oxygen or donor availability, metal toxicity, contaminant accessibility, root stress, or rhizosphere chemistry. In this class, the material is an enabling agent, and the remediation claim depends on direct evidence that biological activity changed the contaminant endpoint. The third is true biologically mediated remediation with a nano component, in which microorganisms or plants perform the risk-relevant transformation, degradation, immobilization, uptake, or exposure interruption, and the nanomaterial only supports delivery, protection, or monitoring (Taha and Mobasser, 2015; Ijaz et al., 2020; Chauhan et al., 2021; Hidangmayum et al., 2023; Cardoso et al., 2025). These distinctions prevent category inflation. A lower extractable concentration after carbonaceous amendment is an adsorption or retention claim unless degradation products, mineralization, toxicity reduction, or isotope evidence show transformation. Lower metal leachability after iron-based amendment is an immobilization claim, not degradation. Improved plant growth after nanoparticle addition is a plant-response claim unless contaminant mobility, tissue burden, biomass fate, or rhizosphere transformation is resolved. PFAS-contaminated soils make the boundary especially clear: reduced pore-water PFAS or leaching after sorbent addition should be reported as immobilization, retention, or exposure control unless destruction, mass balance, transformation products, and residual toxicity are directly demonstrated (Liang et al., 2024). The practical rule is therefore simple: nano-enabled evidence must first be read as a mechanism claim, and only then as a remediation claim.

Recent pesticide-rhizosphere syntheses that combine nano-engineered materials, CRISPR-enabled pathway design, and multi-omics-guided monitoring are relevant to this review only when their claims are kept within this evidence structure. They can support design of pesticide detoxification pathways, target selection, rhizosphere monitoring, and hypothesis testing, but they do not establish field readiness unless contaminant-product chemistry, toxicity, biological activity, ecological containment, and soil-scale durability are documented (Shahid and Touzout, 2026).

The synthesis route should also be kept separate from the remediation mechanism. Biogenic or plant-assisted synthesis may reduce the need for some reagents and may introduce surface coatings that affect particle behavior, but it does not make a nanomaterial safe or field-ready. Green synthesis still requires control of size distribution, surface chemistry, impurities, ion release, reproducibility, toxicity, and environmental fate (Suresh et al., 2011; Ijaz et al., 2020; Ying et al., 2022; Deka et al., 2025). Nanoparticle origin should therefore be reported as material characterization, not used as a claim of environmental compatibility. Chemical notation must also be precise, and surface retention of contaminants should be described as adsorption, complexation, precipitation, or immobilization unless true absorption is demonstrated. Microbial and fungal synthesis studies are used here to define material origin and plausible surface functionalization, not to infer field safety (Vaidyanathan et al., 2010; Suresh et al., 2011; Karthik et al., 2016; Abdelrahim et al., 2017; Mahanty et al., 2019). Broader reviews of agricultural and soil nanotechnology are therefore retained as orientation sources. They are not treated as evidence that nano-enabled remediation is field-ready without endpoint-specific fate, toxicity and monitoring data (Dhanapal et al., 2024; Cardoso et al., 2025; Panigrahi et al., 2025). Plant-assisted and other green-synthesis papers help explain precursor chemistry, surface coatings and synthesis conditions, but they do not remove the need for particle characterization, dose-response testing and fate assessment in soil (Ghosh et al., 2021; Koul et al., 2021; Rai et al., 2021; Tyagi et al., 2021; Ying et al., 2022; Preenanka and Sebastian, 2022; Campaña et al., 2023; Khan et al., 2023; Solís-Sandí et al., 2023).

Mechanism-specific interpretation is essential for nanoscale zero-valent iron (nZVI), TiO_2_, Fe_3_O_4_, carbonaceous nanomaterials, and metal-oxide nanoparticles. nZVI is often described too narrowly as a radical-driven oxidant, whereas in many systems reductive transformation, electron transfer, iron corrosion, surface reactions, hydrogen generation, and passivation control performance (O'Carroll et al., 2013; Dong et al., 2019). TiO_2_ can generate reactive oxygen species (ROS) under irradiation, but light availability, soil opacity, organic matter, particle shielding, and product toxicity limit direct extrapolation from aqueous systems (Zhang et al., 2008; Chauhan et al., 2021). Carbon-based materials may reduce exposure through sorption, yet the same process can reduce microbial access to hydrophobic substrates (Othman and Elsayed, 2022). Fe-based nanoparticles may lower metal mobility, but their value depends on the stability of immobilized fractions under changing pH, redox, wetting-drying cycles, and root-zone chemistry (Gil-Díaz et al., 2019; Gil-Díaz et al., 2020; Gil-Díaz et al., 2022).

The evaluative distinctions are condensed in Table 6. The table is deliberately organized by function and endpoint, rather than by nanomaterial name, because the same material can support different claims under different soil conditions. Short-term nano-assisted degradation studies are interpreted as mechanistic evidence when they report parent decline but not product fate, toxicity or biological compatibility (Fang et al., 2012; Lin et al., 2019; Zielińska et al., 2020). Stabilization and plant-linked nanomaterial studies carry higher decision value only when they connect the intended endpoint with particle behavior and exposure control; otherwise they remain pre-field evidence (Sun et al., 2021; Alvan et al., 2023; Arahangelova and Loukanov, 2024; Pérez-Hernández et al., 2024). Nano-phytoremediation requires particular caution because the same observation can mean opposite things. Increased metal uptake is favorable for phytoextraction only when harvested biomass is removed and managed safely. Decreased translocation is favorable for phytostabilization only when the objective is lower exposure rather than contaminant removal. Improved plant growth is not sufficient evidence for either endpoint. It may reflect reduced stress, dilution of tissue concentrations, or altered contaminant partitioning. The claim must therefore specify whether the intended outcome is extraction, stabilization, rhizosphere transformation, or plant tolerance. Without that distinction, nano-phytoremediation becomes an ambiguous label rather than a testable remediation strategy (Martínez-Fernández et al., 2015; Huang et al., 2018; Sun et al., 2020; Zand and Tabrizi, 2021; Azeez et al., 2024). Nano-phytoremediation studies are interpreted by endpoint and dose-response, because nanoparticle-enhanced uptake, reduced uptake and plant stimulation represent different remediation claims (Gao and Zhou, 2013; Jiamjitrpanich et al., 2013; Ma et al., 2013; Oyelami and Semple, 2015). Plant-response studies are therefore useful only when plant performance is connected to rhizosphere transformation, contaminant partitioning, biomass management or exposure control, not when stimulation is reported as a stand-alone success marker (Pillai and Kottekottil, 2016; Fellmann and Eichert, 2017; Romeh and Saber, 2020; Zand and Tabrizi, 2021).

**TABLE 6:** Mechanism-based interpretation of nano-enabled remediation in soil.

Nanomaterial function	Valid endpoint	Evidence required	Main residual risk	References
Reductive transformation	Dechlorination, Cr(VI) reduction, altered speciation	Parent-daughter products, redox status, pathway completion	Incomplete transformation, passivation, secondary mobilization	O'Carroll et al. (2013), Gil-Díaz et al. (2022)
Adsorption or complexation	Lower pore-water concentration, leachability, or bioavailability	Pore-water data, desorption, leaching, toxicity	Apparent removal without degradation or durable risk reduction	Taha and Mobasser (2015), Othman and Elsayed (2022), Akhtar et al. (2024)
Photocatalysis or ROS-mediated oxidation	Transformation of selected organic pollutants	Irradiation conditions, products, toxicity, soil-shielding assessment	Toxic intermediates, limited light penetration, biotic oxidative stress	Zhang et al. (2008), Chauhan et al. (2021)
Metal immobilization	Reduced mobility, leachability, or plant availability	Speciation, pore-water metal, pH, Eh, stability testing	Remobilization under changed pH, redox, or root chemistry	Gil-Díaz et al. (2019), Gil-Díaz et al. (2020), Gil-Díaz et al. (2022)
Nano-phytoremediation support	Phytoextraction or phytostabilization, defined before treatment	Biomass, tissue burden, translocation, leachability, biomass handling	Contaminated biomass, trophic exposure, endpoint confusion	Huang et al. (2018), Sun et al. (2020), Azeez et al. (2024)
Biological support or stressor	Preserved or enhanced microbial and plant function	Microbial biomass, enzyme activity, plant stress markers, toxicity	Loss of soil function despite contaminant decline	Gómez-Sagasti et al. (2019), Metryka et al. (2022), Chen S. et al. (2024)

Eh, redox potential; ROS, reactive oxygen species.

The strongest constraint on nano-enabled remediation is biological compatibility. Nanoparticles are not inert carriers in soil. They may alter microbial membranes, respiration, ATP production, oxidative stress response, enzyme activity, community structure, nitrification, carbon cycling, root elongation, photosynthesis, and plant oxidative status (Ben-Moshe et al., 2013; Grün and Emmerling, 2018a; Grün et al., 2018b; Gómez-Sagasti et al., 2019; Metryka et al., 2022; Metryka et al., 2023; Chen S. et al., 2024; Metryka et al., 2024). This does not justify a blanket rejection of nanomaterials. It does mean that a nano-enabled treatment cannot be called biological remediation if it reduces the target contaminant while damaging the microbial or plant functions required for soil recovery. The minimum claim should be narrower: chemical transformation or stabilization occurred under specified conditions, with biological compatibility demonstrated only when it has been directly measured.

**FIGURE 3:**
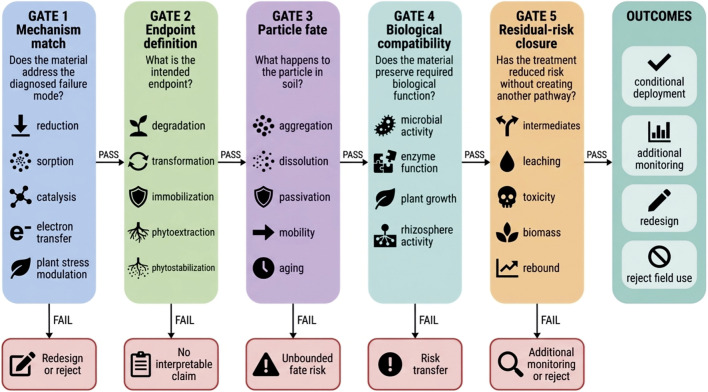
Risk-gated framework for nano-enabled soil remediation. The framework treats nanomaterials as reactive intervention agents, not default enhancers of bioremediation. Field use is admissible only when the material matches a diagnosed limitation, the endpoint is defined, particle fate is bounded, biological compatibility is measured, and residual risk is acceptable. Failure at any gate should lead to redesign, additional monitoring, or rejection of deployment.

The critical issue is not whether toxicity occurs in every soil, but whether the study has measured the functions that the treatment depends on. If the target mechanism is microbial degradation, loss of respiration, enzyme activity or community function weakens the claim even when chemical removal improves. If the target mechanism is phytostabilization, root toxicity or altered translocation can invalidate the endpoint. Plant-toxicity studies are used to bound this risk where nanoparticles alter root growth, gas exchange, photosynthesis or oxidative stress, because these responses can undermine phytoremediation even when pollutant measurements improve (Wang et al., 2016; Fellmann and Eichert, 2017; Abdelsalam et al., 2018; Rajput et al., 2018a; Rajput et al., 2018b; Chung et al., 2019). Soil-function and genotoxicity studies provide a separate warning: changes in enzymatic activity, nitrification, microbial structure or genotoxic endpoints can turn an apparent contaminant benefit into risk transfer (Tilston et al., 2013; Simonin et al., 2018; Fedorenko et al., 2021; Juárez-Maldonado et al., 2021; Liu et al., 2021; Shad et al., 2021; Afzal and Singh, 2022; Sun et al., 2022). The gating logic for field interpretation is summarized in Figure 3. This gate structure prevents a direct jump from laboratory efficiency to field readiness.

Accordingly, nano-enabled treatment is admissible only as a mechanism-matched escalation with bounded particle fate, verified biological compatibility where a biological claim is made, and residual-risk control.

## 4 Omics evidence in soil bioremediation: from genetic potential to verified pathway operation

Omics technologies have changed how soil bioremediation is studied, but their interpretive value is often overstated. They do not remediate soil. They provide evidence about biological potential, activity, stress, interaction, and pathway operation. The critical issue is not whether metagenomics, metatranscriptomics, metaproteomics, or metabolomics are useful, but what kind of claim each data layer can support. A gene indicates capacity. A transcript indicates recent expression under the sampled condition. A protein indicates that part of the biochemical machinery is present. A metabolite may indicate pathway activity, stress, root exudation, or abiotic transformation. None of these layers alone proves causal contaminant removal, detoxification, or field closure (Villas-Bôas and Bruheim, 2007; Ufarté et al., 2015; Sharma et al., 2022). This distinction is central because the literature frequently moves too quickly from molecular association to functional conclusion. A shift toward hydrocarbon-associated taxa does not prove hydrocarbon degradation by those taxa. Detection of catabolic genes does not prove expression. Expression of a pathway gene does not prove flux through the full pathway. Detection of a protein does not prove that the enzyme controlled the observed contaminant kinetics. Detection of an intermediate does not identify its biological source or establish that toxicity declined. These limitations are not arguments against omics. They define the conditions under which omics becomes decision-relevant. The most useful molecular studies are those that are temporally aligned with the contaminant process. A transcript signal collected after the active phase has passed may miss the pathway, while a metabolite measured without a time series may represent accumulation rather than flux. Omics evidence should therefore be interpreted with sampling time, soil fraction, treatment stage and chemical trajectory in view.

Isotope-based approaches should be separated from ordinary omics because they address a different inferential gap. Compound-specific isotope analysis (CSIA) can help distinguish contaminant transformation from dilution, volatilization, sorption, extraction artifacts, or redistribution by measuring isotope fractionation in the target compound. Its strength is reaction attribution; its limitation is that it does not identify the organisms responsible for the process. SIP addresses the biological side of that problem by tracing labeled substrate or contaminant-derived atoms into DNA, RNA, proteins, lipids, or metabolites of active organisms. SIP can therefore link substrate use to active populations or functions, but incubation artifacts, substrate-dose effects, cross-feeding, and matrix disturbance must be controlled. CSIA and SIP are complementary rather than interchangeable: CSIA asks whether transformation occurred, whereas SIP asks which organisms or functions were active during substrate assimilation or transformation. The strongest biological-attribution claims arise when isotope evidence is aligned with parent-daughter chemistry, redox conditions, activity-linked molecular data, toxicity endpoints, and post-treatment monitoring.

Metagenomics is strongest when the uncertainty is genetic capacity. It can identify candidate taxa, catabolic genes, resistance traits, biosurfactant-related pathways, and potential metabolic networks in contaminated soils (Ufarté et al., 2015; Eze, 2021; Huang et al., 2021; Galisteo et al., 2024; Mariano et al., 2024). Its weakest use is as direct evidence of activity. Soil DNA includes signals from active cells, dormant cells, dead cells, extracellular DNA, and poorly characterized organisms. Amplicon profiles add another constraint because they are compositional: an apparent increase in one group may partly reflect a decrease in another rather than true growth (Carini et al., 2016; Gloor et al., 2017). Thus, DNA-based data are most valuable when they define what a soil microbiome could do, not what it demonstrably did during remediation. Reviews and soil-specific sequencing studies are treated as capacity evidence when they identify community change, functional genes or candidate degraders without resolving pathway chemistry (Galitskaya et al., 2021; Melekhina et al., 2021; Wang et al., 2022). They carry greater interpretive weight when sequence data are connected to experimental validation, toxicity or contaminant transformation rather than to taxonomic description alone (Alidoosti et al., 2024; Nagy et al., 2024). Microcosm evidence from related matrices can inform ecological-risk and monitoring design, but transfer to field soil remains conditional unless soil structure, exposure pathway and activity endpoints are preserved (Chen J. et al., 2024). This is why molecular data should rarely be the highest line of evidence in a closure argument. It can strengthen a mechanistic claim, reveal a missing pathway or show stress after treatment, but it should be subordinated to contaminant kinetics, intermediates and toxicity when the decision concerns closure.

Expression and protein-level methods move the inference closer to activity, but they do not remove the need for chemistry. Metatranscriptomics and reverse-transcription quantitative polymerase chain reaction (RT-qPCR) can show whether target pathways respond to contamination, inoculation, plant roots, or amendments (Pacwa-Płociniczak et al., 2020; Čaušević et al., 2024; Pacwa-Płociniczak et al., 2024). Metaproteomics can provide enzyme-level evidence, but soil remains a difficult matrix because proteins bind to minerals and organic matter, humic substances interfere with extraction, and peptide assignment depends on reference databases (Bastida et al., 2009; Bastida et al., 2016; Jouffret et al., 2021; Lee et al., 2022; Li L. et al., 2024). These methods are strongest when paired with contaminant kinetics, daughter-product profiles, electron donor and acceptor trends, and toxicity data. Without that pairing, they support biological response, not remediation success. Absolute abundance also matters. Relative gene or transcript profiles can suggest selection, but they can hide biomass loss, dilution or compositional artifacts. When molecular evidence is used to support remediation, it should be paired with microbial biomass, gene copy numbers, internal standards or another quantitative anchor that prevents relative data from being mistaken for process rate.

Metabolomics has a different strength. It can expose pathway bottlenecks, stress chemistry, rhizosphere signals, and possible transformation products. This makes it particularly useful when parent-compound decline may conceal toxic intermediate accumulation. Yet soil metabolomics is also prone to source ambiguity. Metabolites can be produced by plants, bacteria, fungi, fauna, abiotic reactions, or contaminant transformation; they can also adsorb to minerals, turn over quickly, or remain only tentatively annotated in mass-spectrometry workflows (Villas-Bôas and Bruheim, 2007; Jones et al., 2013; Li et al., 2020; Feng et al., 2024). For this reason, metabolomics should be interpreted through time-series data, authentic standards for key compounds, internal controls, contaminant chemistry, and toxicity endpoints. The strongest metabolomic use is therefore targeted rather than purely exploratory. Untargeted profiles can generate hypotheses, but closure-relevant interpretation requires attention to known intermediates, authentic standards for key metabolites, matrix recovery and toxicity. Otherwise, metabolomics risks replacing one ambiguous signal, parent-compound loss, with another, a partially annotated chemical feature.

**FIGURE 4:**
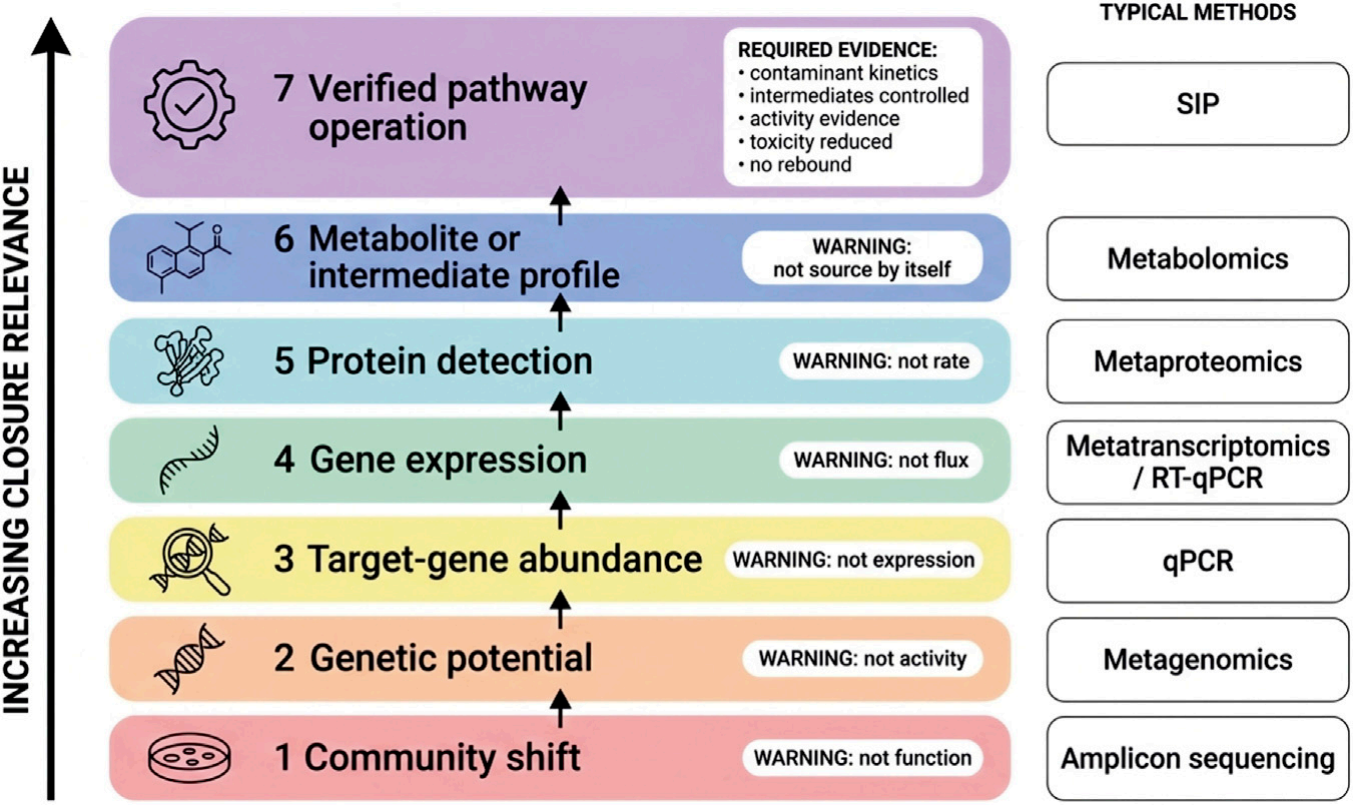
Evidence ladder for omics-supported soil bioremediation. The ladder ranks molecular evidence by its distance from a closure-relevant claim. Community shifts, genes, transcripts, proteins, and metabolites can support diagnosis or monitoring, but they do not by themselves establish pathway operation. The strongest inference requires convergence among contaminant kinetics, intermediate control, activity-linked biological evidence, toxicity reduction, and post-treatment stability. qPCR, quantitative polymerase chain reaction; RT-qPCR, reverse-transcription quantitative polymerase chain reaction; SIP, stable isotope probing.

Table 7 separates each molecular or isotope-linked layer by the claim it can support, and Figure 4 ranks those claims by distance from closure. Omics should be used backward from the decision question: genetic potential can support diagnosis, expression and protein evidence can support activity, isotope-linked and metabolite evidence can strengthen pathway attribution, and toxicity plus post-treatment monitoring remain necessary for closure. Molecular evidence should therefore function as validation and falsification, not as a stand-alone endpoint. This interpretation is supported by independent limitations across the omics layers: compositional distortion in marker-gene data, incomplete activity inference from DNA, extraction and database constraints in metaproteomics, and source ambiguity in soil metabolomics (Gloor et al., 2017; Jouffret et al., 2021; Lee et al., 2022; Feng et al., 2024; Li L. et al., 2024; Cycoń, 2026c). Omics can make a biological explanation plausible, direct targeted monitoring, or show whether an intervention shifted the active community toward a required pathway. It should not decide closure without convergence among contaminant kinetics, expected products, activity-linked biological evidence, toxicity reduction, and post-treatment stability. The next challenge is whether designed microbial systems, including SynComs, can retain their intended function after soil imposes ecological selection.

**TABLE 7:** Omics inference in soil bioremediation: evidence strength and required validation.

Method	What it can support	Main limitation	Required validation
Amplicon sequencing	Community shift and ecological selection	Relative data, primer bias, weak functional inference	Absolute quantification, functional markers, chemistry
Metagenomics	Catabolic potential and resistance traits	DNA does not prove activity	Transcripts, proteins, metabolites, pathway chemistry
RT-qPCR and metatranscriptomics	Recent expression of selected or community-wide genes	Expression does not prove flux	Time series, parent-daughter products, activity assays
Metaproteomics	Enzyme-level evidence of biological machinery	Extraction bias and database limits	Enzyme activity, metabolites, contaminant kinetics
Metabolomics	Intermediates, stress signals, rhizosphere chemistry	Source ambiguity and matrix effects	Standards, temporal profiles, toxicity endpoints
CSIA	Transformation attribution and separation of biodegradation from dilution, sorption, volatilization, or redistribution	Requires interpretable isotope fractionation, sufficient concentration, pathway knowledge, and control of transport effects	Parent-daughter chemistry, isotope enrichment factors where available, geochemistry, mass balance, and toxicity endpoints
SIP	Linkage between contaminant-derived or substrate-derived atoms and active organisms or functions	Incubation artifacts, high substrate dose, cross-feeding, and disturbance of native soil conditions	Field-relevant dosing, time series, chemical endpoints, activity markers, and toxicity endpoints

CSIA, compound-specific isotope analysis; RT-qPCR, reverse-transcription quantitative polymerase chain reaction; SIP, stable isotope probing.

## 5 Synthetic microbial communities: designed function under ecological selection

Synthetic microbial communities (SynComs) occupy a difficult position in soil bioremediation. They promise more control than undefined consortia and more functional breadth than single-strain bioaugmentation, but this promise is often inferred from design rather than demonstrated after soil exposure. A SynCom should be defined as a known set of organisms assembled to test, reproduce, or direct a specified function. Its defining features are membership, assigned roles, and measurable function, not simply the fact that strains were co-cultured before application (Großkopf and Soyer, 2014; McCarty and Ledesma-Amaro, 2019; Li M. et al., 2024; Mehlferber et al., 2024). This definition matters because the critical unit of evidence is not the inoculum at time zero. It is the function retained after the community enters a heterogeneous, competitive, and chemically stressful soil environment.

The strongest argument for SynComs is mechanistic complementarity. Many remediation endpoints require more than one biological role: primary degradation, intermediate consumption, pH buffering, biosurfactant production, stress tolerance, plant-growth support, metal resistance, or root colonization. A designed community can, in principle, distribute these roles across members. This is a real conceptual advance over single-strain bioaugmentation, where one organism is expected to survive, disperse, compete, express the target pathway, and complete the endpoint. Yet complementarity is not established by adding more strains. It must be shown through a functional logic: which member performs which role, which interaction is required, and what failure would occur if that member or interaction were lost. This makes falsifiability the core design criterion. A credible SynCom design should specify the expected loss of function when a member is removed, a substrate exchange is blocked, or a stress-buffering role fails. Without that counterfactual, the community is only a mixture with a favorable outcome, not a designed system with a tested mechanism. Cross-feeding evidence is weighted more strongly than simple co-inoculation because it provides a testable mechanism for community-level performance (Liu et al., 2019; Xu et al., 2019). Interaction-specific and newer SynCom studies carry additional value when they test whether the designed relation persists under altered resources, stress, or community context (Fritts et al., 2021; Liu et al., 2023; Wu et al., 2025).

This requirement changes how the literature should be weighed. Network-based design, genome-scale modeling, natural-community simplification, and top-down plus bottom-up assembly can all help identify candidate SynComs (Toju et al., 2020; Gonçalves et al., 2023; Ruan et al., 2024; Thieffry et al., 2024; Xu and Jiang, 2024). These approaches are valuable because they move community construction beyond intuition. Their limitation is the same across methods: a predicted interaction is not a field-stable interaction. Association networks do not prove causality. Metabolic models depend on assumptions about substrates, exchange, and environmental constraints. A community that degrades a pesticide in one soil may fail when moisture, pH, organic matter, resident microbiota, or contaminant aging changes. SynCom studies are therefore strongest when they combine design with real-soil testing, strain-resolved monitoring, activity evidence, contaminant kinetics, and assessment of intermediates or toxicity. Earlier synthetic-biology literature is used here to define trait logic and controllability, not as direct evidence of field remediation (Barriault et al., 2002; Roggo et al., 2013; Church et al., 2014). More recent design-oriented studies are useful for framing engineered consortia, but their claims should be limited to design space, trait structure, or experimental control until retained function is shown in soil. Community-engineering papers help specify design concepts such as assembly, functional redundancy, and ecological constraint, but they remain pre-field evidence unless connected to soil testing and endpoint monitoring (de Souza et al., 2019; Jaiswal et al., 2019; Lawson et al., 2019).

Ecological drift is the central field problem for SynComs. Once a designed community is introduced into soil, its composition and function are exposed to habitat filtering, nutrient competition, pore-scale separation, predation, phages, dormancy, dispersal constraints, plasmid loss, and interaction with native microbiota. The designed ratio of members may change quickly, and loss of a low-abundance helper can matter more than loss of a numerically dominant member if that helper removes a toxic intermediate or supports a limiting metabolic exchange. This is why persistence alone is insufficient. A SynCom can persist but lose function, or lose part of its membership while the native microbiome takes over the intended process. The decisive question is whether the endpoint remains attributable, safe, and stable after ecological filtering (Mawarda et al., 2020; Čaušević et al., 2024; Papin et al., 2024; Chen C. et al., 2025). Propagation studies support this caution because reproducibility before release does not remove habitat filtering after release (Čaušević et al., 2022). Transplant or community-transfer evidence is more informative when it shows which functions persist, which are replaced by the resident microbiome, and which fail under the receiving soil conditions (Čaušević et al., 2025).

Plant-linked SynComs require the same discipline. Improved plant growth, stress tolerance, or root colonization should not be interpreted automatically as remediation success. These responses may support remediation only when they are connected to a predefined endpoint, such as phytoextraction, phytostabilization, rhizodegradation, or soil-function recovery. In metal-contaminated systems, higher uptake may be desirable for extraction but undesirable for stabilization. In organic-contaminant systems, stronger rhizosphere activity is relevant only if parent-compound decline, intermediate control, and toxicity reduction are shown. Thus, plant-associated SynComs should be judged by the contaminant endpoint and biomass or exposure management, not by plant performance alone (Gkorezis et al., 2016; Pacwa-Płociniczak et al., 2023; Huang et al., 2024). Plant-linked functional communities are interpreted through contaminant endpoints and spatial control rather than through plant response alone (Shi et al., 2023). Targeted root-colonization systems add decision value only when colonization is connected to the active contaminant zone and to a defined remediation endpoint (Wu et al., 2024).

CRISPR-based editing and related synthetic-biology tools can make pathway testing more specific, but they also increase the burden of evidence. For pesticide detoxification, edited strains or engineered consortia should be evaluated by parent-compound loss, regulated metabolites, suspect transformation products, toxicity, persistence of the intended function after soil exposure, horizontal gene-transfer risk where relevant, and containment. The pesticide-rhizosphere framework proposed by Shahid and Touzout (2026) is therefore useful as a design and monitoring source for nano-enabled and CRISPR-enabled detoxification strategies. In this review, however, it is treated as design-level and validation-level evidence, not as a field-closure precedent.

A further constraint is product identity. A designed community cannot be assumed to remain the same product after storage, transport, carrier release and early soil selection. Pre-field evidence should therefore include not only the initial composition but also stability during formulation, viability after delivery, and functional recovery after contact with the target soil. Recent reviews of microbial degradation and engineered SynCom applications are useful for framing this design space, but they do not lower the requirement for soil testing and monitoring of retained function (Liu et al., 2025). SynCom work focused on soil and plant resilience is relevant to this review only when plant performance is kept separate from remediation success. Member diversity, functional complementarity, and stress buffering can support phytoremediation design, but increased plant vigor is not a closure endpoint. In contaminated soil, such evidence becomes decision-relevant only when it is linked to contaminant mobility, tissue burden, rhizosphere transformation, phytostabilization, exposure control, biomass fate, or post-treatment soil function (Tariq et al., 2025).

For field deployment, a SynCom should be treated as a biological product, not merely as an experimental design. The minimum pre-field evidence should include known membership, assigned function for each member, interaction testing, formulation, carrier selection, shelf life, dose, delivery route, strain tracking, activity markers, contaminant endpoints, and secondary-risk assessment. These requirements are not procedural excess. They are the conditions under which the claim “designed community” becomes more than a laboratory label. Without them, SynComs risk repeating the central weakness of conventional bioaugmentation: strong biological promise under controlled conditions, followed by weak functional evidence after entry into soil. SynComs therefore have a legitimate but bounded place in soil bioremediation. They are most defensible when the diagnosed failure mode is biological interaction itself: missing downstream metabolism, toxic intermediate accumulation, weak rhizosphere support, metal stress limiting plant function, or unstable degradation by single strains. They are least defensible when the primary limitation is geochemical, physical, or endpoint-related. A designed community cannot solve poor oxygen delivery, contaminant sequestration, redox mismatch, or undefined closure criteria by design alone. Under a critical-review standard, SynComs should be viewed as high-value experimental and early deployment platforms, not as general field-ready solutions. Their credibility will depend on whether designed function can be retained, measured, and linked to risk reduction after soil imposes selection. This leads directly to the next evidentiary layer: AI can help design, predict, and monitor complex systems, but only if its data domain, validation, and decision claim are explicit.

## 6 Artificial intelligence in soil bioremediation: decision support under field-data scarcity

Artificial intelligence (AI) should be treated as a decision-support layer, not as a remediation mechanism. This distinction is essential because current AI applications in soil remediation mostly predict, classify, optimize, extract, or rank information. They do not demonstrate degradation, detoxification, immobilization, plant uptake, or closure by themselves. A model may estimate polycyclic aromatic hydrocarbon (PAH) concentration, total petroleum hydrocarbon (TPH) removal, soil-quality indicators, contaminant distribution, or treatment response with acceptable internal error. That does not prove that the predicted endpoint is causal, durable, safe, or transferable to another soil. The critical question is therefore not whether AI can fit environmental data, but whether the model supports a named remediation decision within a validated domain (Olawoyin, 2016; Sanusi et al., 2016; Bao et al., 2019; Anifowose and Anifowose, 2024). Broad AI and digital-biology papers are retained only to frame methodological possibilities, not to support claims of remediation performance (Zhang and Ma, 2012; Nielsen and Voigt, 2018; Khosravi et al., 2021). Recent AI-oriented reviews and applications are therefore treated as low-decision-weight unless they report the training domain, validation design, and decision consequences (Adetitun et al., 2025; Rosca and Stancu, 2025). Monitoring, text-mining, and remediation-review papers are used as context when they clarify data extraction, observation systems, or model architecture, but they do not replace validation against a named soil endpoint (Blanchy et al., 2023; Patowary et al., 2023; Akintola, 2024; Ashkanani et al., 2024; Vaughan et al., 2024).

The strongest current role of AI is bounded prediction under clearly specified conditions. It can help prioritize sampling, estimate contaminant patterns, compare operational settings, screen treatment options, extract variables from the literature, or predict possible transformation products. These uses are scientifically useful when they reduce uncertainty that would otherwise affect a field decision. They are weak when the model predicts a proxy and the proxy is then treated as remediation evidence. Electrical conductivity, vegetation stress, predicted PAH concentration, or modeled TPH decline can guide interpretation, but none is equivalent to toxicity reduction, pathway completion, metal stabilization, or safe closure. Decision-grade digital twins occupy a narrower but important position in this hierarchy: they can formalize the conceptual site model, combine chemistry, microbiology, hydraulics, delivery, monitoring triggers, uncertainty, and closure logic, but they remain decision-support systems rather than treatment evidence (Cycoń, 2026b). This is the same evidentiary problem seen with omics: a signal may be informative, but its claim must not exceed its validation.

Most AI studies in this area face a data problem before they face an algorithm problem. Soil datasets are often small, spatially dependent, chemically heterogeneous, and collected under different extraction, detection, and reporting protocols. Treatment endpoints are also inconsistent. Some studies predict parent-contaminant decline, others predict plant tissue burden, physicochemical indicators, microbial profiles, or risk categories. These endpoints are not interchangeable. A model trained on controlled experiments or pot systems may perform well inside that dataset but fail when applied to aged contamination, mixed pollutants, different soil texture, altered moisture, or field-scale treatment delivery. Spatial or temporal dependence can also inflate performance when nearby samples occur in both training and test sets (Roberts et al., 2017; Meyer and Pebesma, 2021).

The most common hidden failure is endpoint substitution. A model trained to predict an indicator may be mathematically sound and still irrelevant to remediation if the indicator does not alter the decision. Predicting a concentration surface, plant stress index or soil-quality proxy is useful only when the model states what field action follows from the prediction and what independent evidence would falsify it. For this reason, validation design matters more than model class. Artificial neural networks, random forests, support vector regression, regression trees, Bayesian models, and deep learning methods can all generate useful predictions if the task is well bounded. They can all mislead if validation is weak. Random train-test splits are insufficient when samples are clustered by site, depth, treatment, or time. External validation, blocked spatial or temporal validation, uncertainty reporting, and an explicit applicability domain should be treated as minimum requirements, not optional additions. A model that lacks these elements should be used for hypothesis generation or sampling design, not for field deployment or closure. Applicability domain should be stated in practical terms, not only as a statistical score. A user should know whether the model applies to aged or freshly spiked soil, which contaminant class is represented, which extraction method generated the endpoint, and whether predictions are interpolation within known conditions or extrapolation into a new site. Probabilistic decision models can improve transparency when uncertainty and decision consequences are explicit, but they remain decision aids rather than remediation evidence (Carriger and Parker, 2021). Risk-screening studies add value when they identify which uncertainty would change a decision; they are weaker when they convert sparse environmental data into an apparently precise ranking (Mentzel et al., 2022; Cycoń, 2026b).

AI-linked databases and pathway tools require the same restraint. Resources such as enviPath, Eawag-Soil, the US EPA CompTox Chemicals Dashboard, ECOTOX, and the NORMAN Suspect List Exchange can help identify candidate transformation products, chemical properties, toxicological endpoints, or suspect contaminants (Wicker et al., 2016; Latino et al., 2017; Williams et al., 2017; Olker et al., 2022; Taha et al., 2022; Hafner et al., 2024). Their functions are different and should not be mixed. A pathway-prediction system can suggest what products to monitor, but cannot prove that those products form in a given soil. A toxicity database can support endpoint selection, but cannot replace site-specific bioavailability or mixture testing. A suspect-screening list can improve chemical identification, but cannot determine remediation feasibility. The value of these resources lies in structuring hypotheses and monitoring plans, not in certifying treatment success.

AI also intersects with omics and SynCom design, but this intersection should remain hypothesis-generating unless validated experimentally. Machine learning may help identify candidate degraders, rank microbial interactions, infer functional networks, or prioritize community combinations. These outputs can guide SynCom construction and molecular monitoring. They do not prove that the selected organisms will persist, interact, or function after release into soil. The same applies to AI-assisted interpretation of metagenomes, transcriptomes, proteomes, or metabolomes: prediction can organize high-dimensional data, but causal attribution still requires contaminant kinetics, activity evidence, pathway chemistry, and, when relevant, toxicity reduction.

A minimum reporting standard is therefore necessary for AI claims in soil bioremediation. Each study should state the decision claim, contaminant class, soil type, experimental or field scale, input variables, sample size, spatial and temporal design, preprocessing steps, missing-data handling, model class, validation strategy, error metrics, uncertainty, applicability domain, and practical consequence. It should also state what the model cannot decide. A model predicting PAH concentration cannot decide detoxification. A model optimizing aeration cannot decide rebound. A model classifying soil quality cannot decide whether a metal has been stably immobilized. A model predicting SynCom performance cannot decide ecological persistence without monitoring after application. AI applications addressing pollutant screening and per- and polyfluoroalkyl substance risk are retained as decision-support context, not as direct evidence of remediation success (Adamopoulos et al., 2025; Malwe et al., 2025). Applications focused on enzyme prediction or crop-metal prediction are useful only when their outputs are connected to experimental validation, exposure pathways, or intervention choice (Zhao et al., 2025). Table 8 applies the AI reporting criteria used in this review to representative studies cited in the AI section. It does not rank model complexity. Its purpose is to distinguish models that support bounded prediction or contextual interpretation from models that can support field decisions within a defined applicability domain.

**TABLE 8:** Decision relevance of representative AI-linked studies in soil remediation.

Study	AI claim	Evidence domain	Decision relevance
Olawoyin (2016)	BP-ANN prediction of PAH bioremediation	Soil PAH dataset; internal validation only	Prediction only; no closure claim
Sanusi et al. (2016)	ANN/RSM optimization of TPH phytoremediation	Pilot diesel-soil system; internal optimization	Pilot treatability evidence
Bao et al. (2019)	BP-ANN prediction of PAH after corn straw amendment	Aged PAH soil; internal validation; concentration endpoint	Bounded concentration prediction
Anifowose and Anifowose (2024)	AI/ML for pollution impact and EIA	Review/conceptual domain; no primary validation	Decision-support context
Rosca and Stancu (2025)	AI monitoring and prevention trends	Systematic/bibliometric review; no field model validation	AI application context
Adamopoulos et al. (2025)	AI for PFAS risk and food-supply protection	PFAS risk framework; field validation still needed	Policy and risk context
Zhao et al. (2025)	ANN/MLR prediction of Cd in rice	Guangxi soil-rice field dataset; domain-limited	Exposure prediction only

AI, artificial intelligence; ANN, artificial neural network; BP-ANN, back-propagation artificial neural network; EIA, environmental impact assessment; ML, machine learning; MLR, multiple linear regression; PAH, polycyclic aromatic hydrocarbon; PFAS, per- and polyfluoroalkyl substances; RSM, response surface methodology; TPH, total petroleum hydrocarbons.

AI therefore belongs in diagnosis, sampling design, monitoring, uncertainty analysis, and bounded prediction. It is not a treatment mechanism; intervention selection still depends on the diagnosed failure mode and the evidence required for closure.

## 7 From failure diagnosis to intervention choice: an evidence-weighted pathway

**FIGURE 5:**
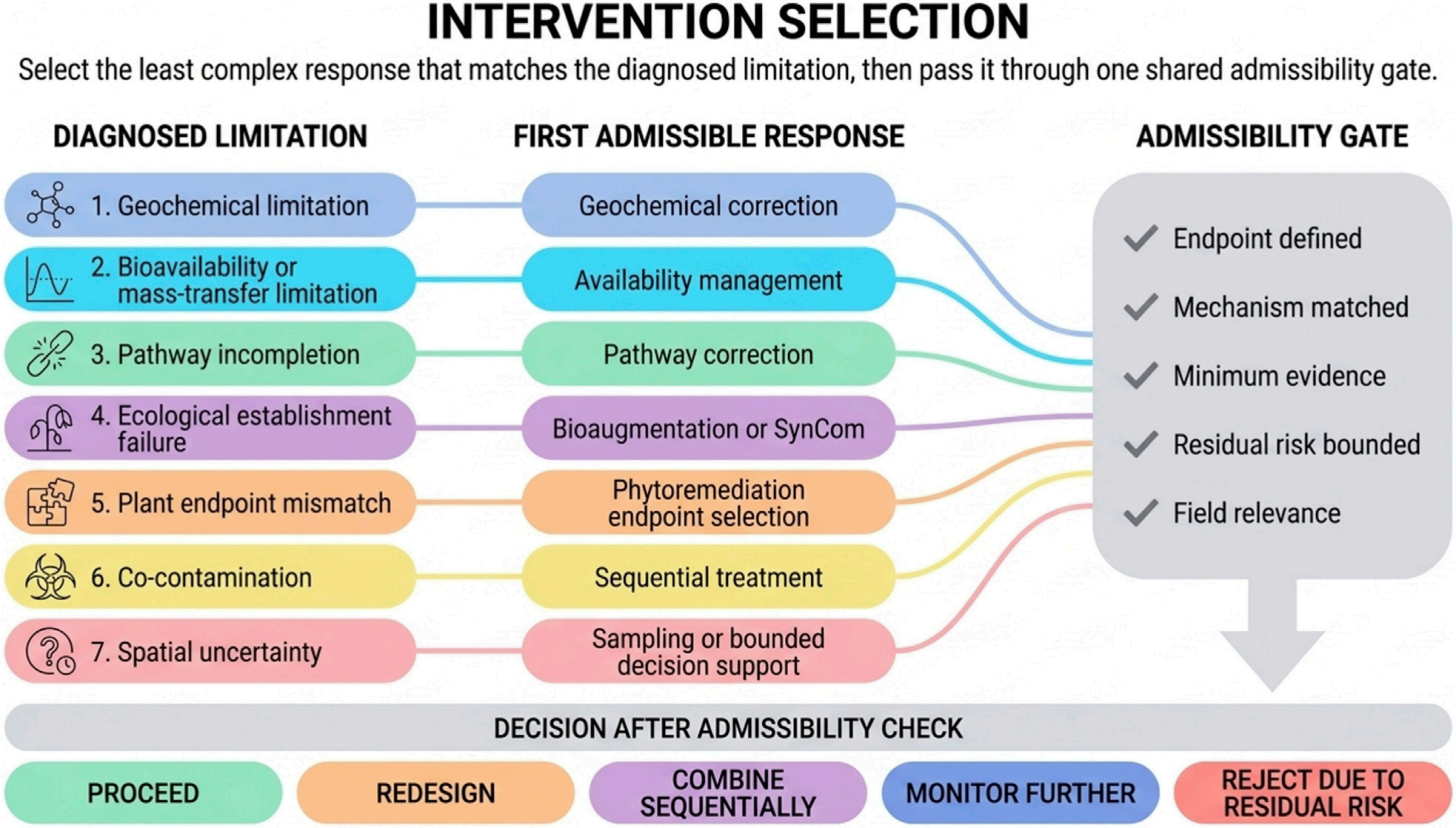
Intervention selection after failure-mode diagnosis. Each diagnosed limitation is linked to the least complex admissible response before escalation. Candidate interventions pass through one shared admissibility gate that requires a defined endpoint, mechanism match, minimum evidence, bounded residual risk, and field relevance. The final decision is to proceed, redesign, combine interventions sequentially, monitor further, or reject the intervention because residual risk remains unacceptable.

This section functions as an admissibility test. Intervention choice should move from the diagnosed limiting process to the least complex treatment able to reach the predefined endpoint, not from technology availability to site design. The evidence reviewed above permits three decisions: proceed with a mechanism-matched correction, escalate only when lower-risk options cannot address the failure mode, or stop and redesign when the endpoint, active fraction, or residual risk cannot be monitored. Table 9 operationalizes this rule; Figure 5 shows the decision sequence.

**TABLE 9:** Failure-mode-to-intervention matrix for evidence-weighted soil bioremediation.

Diagnosed failure mode	First admissible response	Minimum evidence before escalation	Escalation allowed only when	Reject escalation when	Critical endpoint
Nutrient, oxygen, moisture, pH, or redox limitation	Correct delivery conditions first	Depth-resolved oxygen, Eh, moisture, pH, donor/acceptor trends, respiration or pathway indicators	Native capacity remains absent or inactive	Active zone or endpoint is undefined	Parent compound, products, toxicity, activity
Low bioavailability or mass-transfer limitation	Measure accessibility first	Passive sampling, pore-water concentration, desorption kinetics, leaching, toxicity	Release is matched by degradation or exposure control	Release increases risk	Bioavailable fraction, leaching, toxicity
Reductive pathway stall	Rebalance donor and redox	Parent-daughter sequence, *cis*-DCE/*trans*-DCE, vinyl chloride, ethene/ethane, donor and acceptor trends	Terminal function is absent or inactive	Intermediates or donor competition dominate	Ethene formation, toxicity reduction
Co-contamination toxicity	Prioritize dominant toxicity	Metal speciation, pore-water metal, enzyme activity, organic degradation markers, toxicity	Endpoints conflict under single-step treatment	Treatment worsens co-risk	Toxicity, mobility, pathway completion
Toxic intermediate accumulation	Correct pathway bottleneck	Expected products, metabolite time series, pathway markers, toxicity	Downstream metabolism fails	Products or toxicity are unmonitored	Product disappearance, toxicity reduction
Inoculant establishment failure	Optimize inoculum delivery	Strain-specific qPCR/ddPCR, activity markers, contaminant endpoint, post-dosing monitoring	Native function is missing	Persistence lacks function	Function after ecological filtering
SynCom failure or instability	Test member contribution	Member tracking, loss-of-member controls, activity, contaminant endpoint	Interaction corrects failure	Only input composition is measured	Retained function and safety
Phytoremediation endpoint mismatch	Define plant endpoint first	Tissue burden, translocation factor, leachability, rhizosphere activity, biomass fate	Plant response matches endpoint	Growth is the only endpoint	Exposure reduction or biomass-managed extraction
Nano-enabled escalation	Match material to mechanism	Particle identity, dose, fate, leaching, toxicity, microbial or plant compatibility	Material addresses unresolved bottleneck	Fate or compatibility is unmeasured	Risk reduction without risk transfer
AI-supported decision	Use as decision support only	External or blocked validation, uncertainty, applicability domain, decision consequence	Prediction changes a decision	Only calibration is shown	Error-bounded decision support

AI, artificial intelligence; *cis*-DCE, *cis*-1,2-dichloroethene; ddPCR, droplet digital polymerase chain reaction; Eh, redox potential; qPCR, quantitative polymerase chain reaction; SynCom, synthetic microbial community; *trans*-DCE, *trans*-1,2-dichloroethene.

The first decision should usually be conservative. If underperformance results from nutrient imbalance, poor oxygen delivery, moisture limitation, pH stress, or electron donor-acceptor mismatch, geochemical correction should precede biological escalation. Adding degraders to soil where the active zone lacks oxygen, water continuity, or the required redox state does not solve the primary limitation. Similarly, adding omics, AI, nanomaterials, or SynComs to a system with undefined endpoints increases interpretive complexity without improving causal inference. The field has enough evidence to reject automatic escalation. It has less evidence to justify many combined interventions under field conditions (Semple et al., 2003; Perfumo et al., 2007; Diplock et al., 2009; Varjani and Upasani, 2019; Zhang et al., 2020). Conservatism here is not technological caution for its own sake. It is an evidence rule. The intervention with the lowest unaddressed uncertainty should be preferred when it can correct the limiting process. Escalation is justified only when the simpler intervention cannot reach the endpoint or would create a greater risk than the advanced option.

Bioaugmentation and SynComs become defensible only when the biological limitation is demonstrated. If native functional capacity exists but is inactive, biostimulation or redox correction may be more rational than inoculation. If a pathway is absent, incomplete, or ecologically unstable, inoculation can be justified, but only with strain tracking and activity evidence. SynComs require an even narrower rationale: a designed interaction must correct the failure, such as downstream intermediate consumption, cross-feeding, plant stress support, or metal tolerance. Otherwise, a SynCom is merely a more complex inoculum (Vogel, 1996; Mrozik and Piotrowska-Seget, 2010; Mawarda et al., 2020; Čaušević et al., 2024; Cycoń, 2026a).

Nano-enabled treatment should be treated as escalation, not enhancement. It is appropriate when the diagnosed bottleneck is physicochemical and cannot be addressed safely by simpler measures: strong contaminant sequestration, persistent metal mobility, redox transformation requiring reactive surfaces, or plant stress that blocks a defined phytoremediation endpoint. It is not appropriate when its effect is only a short-term decrease in extractable concentration or when particle fate, toxicity, and biological compatibility are unmeasured (Gil-Díaz et al., 2019; Gómez-Sagasti et al., 2019; Chen S. et al., 2024). Omics and AI occupy a different position. They can reduce uncertainty, verify biological activity, prioritize sampling, or define model-supported decisions, but they should not be counted as treatment layers. Their value lies in improving the admissibility of the intervention claim.

Table 9 condenses this hierarchy as an operational escalation filter by specifying the minimum evidence required before a more complex intervention is justified and the conditions under which escalation should be rejected. Figure 5 presents the intervention-selection logic graphically as a simplified sequence from diagnosed limitation to admissible response, shared admissibility check, and decision outcome.

The practical consequence is that integrated remediation should be justified by complementarity, not accumulation. Combining oxygen delivery, nutrients, inoculants, nanomaterials, plants, omics, and AI may be reasonable when each component corrects a distinct failure mode. It is weak when several technologies are assembled because each is current or attractive. A valid integrated strategy can be expressed as a causal sequence: for example, reduce metal bioavailability before expecting organic degradation, correct donor supply before adding a dechlorinating culture, or verify pathway activity before scaling a SynCom. If removing one component from the design would not create a mechanistic gap, that component is not yet justified. Sequential combinations are generally easier to justify than simultaneous combinations because they preserve causal interpretation. If metal toxicity is first reduced, the subsequent organic-degradation response can be interpreted more clearly. If donor addition is adjusted before bioaugmentation, failure of the inoculum is less likely to be misassigned to redox deficiency. Integrated treatment should therefore be staged when staging improves inference. The negative decision is part of field readiness: escalation should be rejected when the endpoint, active fraction, delivery zone, or residual risk cannot be monitored with decision-grade evidence.

## 8 Field readiness, monitoring, and closure: when evidence becomes decision-grade

A soil bioremediation strategy is not field-ready because it performs well in culture, a short incubation, a pot trial, or a real-soil microcosm. Those systems can establish plausibility, mechanism, or treatability. They do not by themselves establish delivery, durability, safety, cost realism, or closure. This distinction is often blurred in the literature. A contaminant may decline during active treatment and rebound after donor supply, aeration, irrigation, amendment activity, plant growth, or inoculant function weakens. A metal may appear immobilized in one extraction fraction and later become more mobile after pH, redox, wetting-drying, organic matter, or root-zone chemistry changes. A model may predict a concentration trend inside the training domain and fail in another soil. Field readiness therefore requires a different evidence standard than laboratory efficacy (Lai et al., 2007; Brusseau et al., 2013; Bardos et al., 2018). The distinction can be expressed as three evidence states. Plausibility means the process can occur. Treatability means the process can occur in relevant soil under controlled conditions. Decision-grade evidence means the process can be delivered, monitored, and judged against the intended land use. Confusing these states is one reason why laboratory success is often narrated as practical readiness.

The first requirement is endpoint clarity. Closure cannot be inferred from “removal” unless the relevant risk has also been reduced. For organic contaminants, this means that parent-compound loss must be interpreted with bioavailable fraction, expected intermediates, toxicity, and, where a biological mechanism is claimed, activity evidence. For chlorinated ethenes, parent-daughter product balance and ethene formation matter more than parent loss alone. For metals and metalloids, total concentration is often secondary to speciation, pore-water concentration, leachability, plant uptake, and exposure. This distinction is especially important for mining-impacted wastes, where oral bioaccessibility and relative bioavailability of arsenic, cadmium, lead, and antimony can diverge from total concentration and materially change risk interpretation (Kastury et al., 2024). For phytoremediation, contaminated biomass is part of the endpoint. For nano-enabled treatment, particle fate and biological compatibility are part of the claim. For SynComs and bioaugmentation, persistence without activity is not enough. These are not optional refinements. They decide whether a treatment reduced risk or only changed the form in which risk is measured. Endpoint clarity also requires rejecting generic language such as “safe,” “restored,” or “cleaned” when the measured variables do not support those claims. A site can meet a chemical threshold while remaining biologically impaired. A stabilized soil can remain unsuitable for extraction. A phytoremediation system can lower soil concentration while creating a contaminated biomass stream. Closure criteria must therefore be named before treatment begins. A further distinction is required between remediation and recovery. Remediation can reduce contaminant pressure, mobility, toxicity, or exposure without restoring the structure and functions of the soil microbiome. Recovery requires evidence that microbial reassembly, process rates, functional redundancy, resistance to renewed stress, and plant-soil interactions have moved toward a condition compatible with the intended land use. This distinction is especially important after aggressive amendments, nano-enabled treatments, repeated donor addition, strong redox manipulation, or engineered microbial release, because contaminant improvement can coexist with impaired soil function (Cycoń, 2026d).

The second requirement is post-intervention evidence. Monitoring only during active treatment favors overly optimistic interpretation because it captures the period when amendments, plants, donor supply, or inoculants are most favorable. Closure should instead ask whether the endpoint persists after active intervention has stopped. Rebound, remobilization, toxic intermediate persistence, loss of microbial function, plant dieback, secondary exposure through biomass, and renewed leaching are all closure failures. A single sampling point immediately after treatment should therefore be treated as weak evidence, regardless of the apparent removal percentage. Monitoring duration should be tied to the failure mode rather than to a fixed calendar. Redox-controlled systems should be followed through donor depletion or electron-acceptor change. Plant-based systems should be followed through growth, senescence, and biomass handling. Stabilization systems should be tested under the pH, redox, and wetting-drying changes likely for the site. The monitoring window is part of the evidence claim.

The third requirement is practical deliverability. A treatment that cannot be applied uniformly, monitored adequately, maintained within cost limits, or accepted under regulatory constraints is not field-ready. This matters especially for technologies that add analytical or operational burdens, including nanomaterials, SynComs, genetically modified organisms, high-frequency omics monitoring, and AI-supported decision systems. Their value must be judged by the decision they improve, not by their technical sophistication. Sustainability assessment follows the same logic: energy use, amendment production, biomass disposal, secondary waste, treatment duration, monitoring intensity, stakeholder exposure, and residual liability are part of the remediation claim, not external commentary (Bardos et al., 2018). Practical deliverability also includes the ability to stop or redirect a treatment. A field-ready design should define failure triggers: rebound beyond an agreed threshold, metabolite accumulation, metal remobilization, loss of activity, unacceptable toxicity, or failure to reach the active zone. Without failure triggers, monitoring becomes descriptive rather than decision-making. Leaching studies can inform exposure-pathway design, but they should not be converted into bioremediation closure evidence without a matching soil-remediation endpoint (Duan X. et al., 2024). Mixed-contaminant monitoring studies add value when they show how chemical fractions, biological response, and exposure change together; otherwise they remain supporting evidence for monitoring design rather than proof of closure (Duan Y. et al., 2024).

**FIGURE 6:**
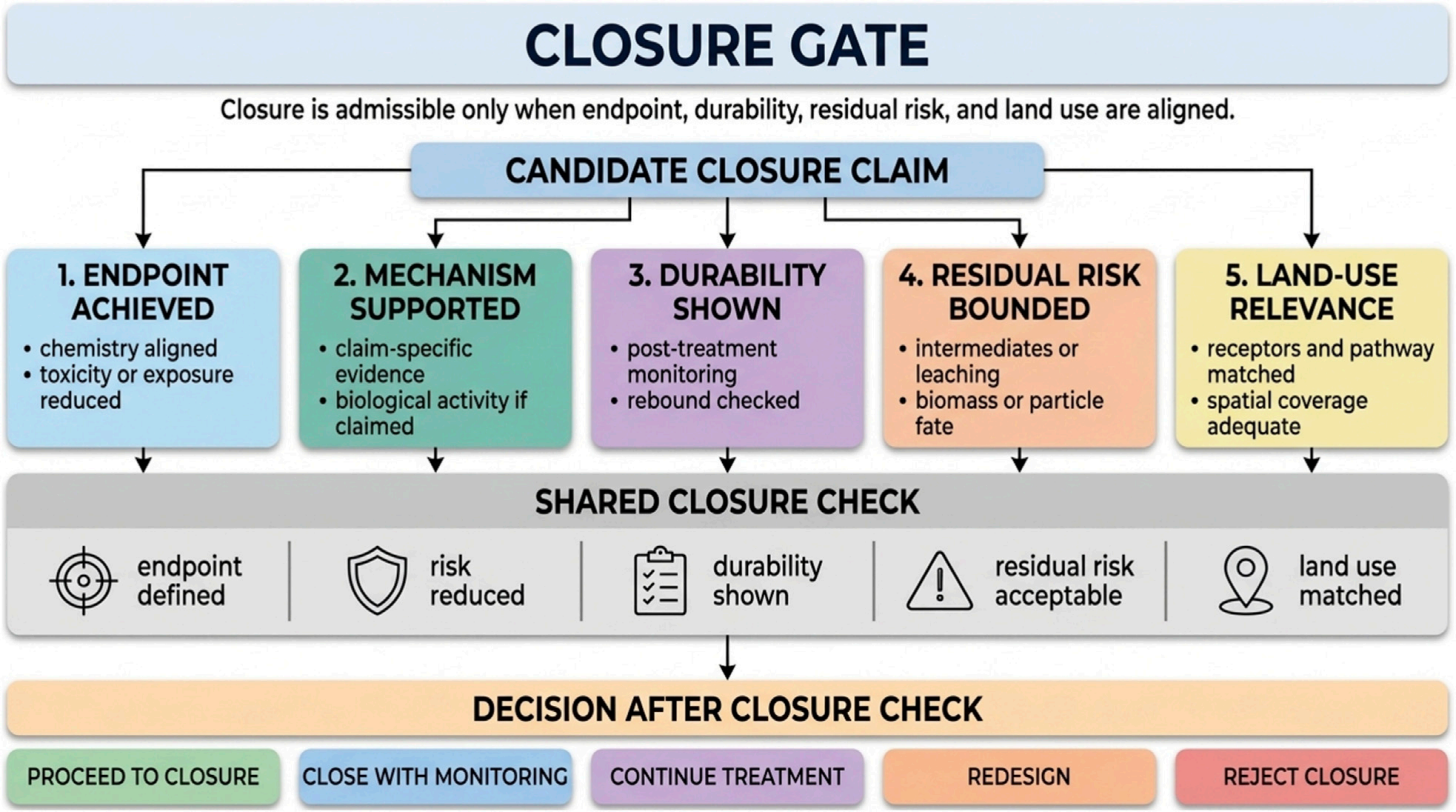
Closure gate for soil bioremediation and integrated remediation strategies. The figure treats closure as a candidate evidence claim rather than a removal percentage. Closure is admissible only when the endpoint has been achieved, the mechanism is supported, durability has been shown, residual risk is bounded, and the result is relevant to the intended land use. The shared closure check integrates endpoint definition, risk reduction, durability, residual-risk acceptability, and land-use match before the final decision to proceed to closure, close with monitoring, continue treatment, redesign, or reject closure.

Table 10 is the final field-deployment and closure checklist. It deliberately avoids repeating the evidence rubric in Table 2 or the escalation matrix in Table 9; its function is to separate what must be known before field deployment from what must be shown before closure. Figure 6 then presents the same logic as a gate sequence. The central judgment is that closure should be designed backward from the final decision. If the decision concerns safe land reuse, the evidence must address exposure and residual function, not only contaminant concentration. If the decision concerns stabilization, the evidence must address mobility and durability, not total concentration alone. If the decision concerns biological remediation, activity must be verified rather than inferred from presence. Field-ready bioremediation is therefore not defined by the complexity of the technology used, but by the strength of the evidence connecting diagnosis, intervention, endpoint, durability, and residual risk. This standard sets the basis for the final conclusion: the field will advance only if it treats bioremediation as a staged evidence claim rather than a technology label.

**TABLE 10:** Field-readiness and closure package for soil bioremediation studies.

Domain	Required before field deployment	Required before closure	Closure failure trigger
Conceptual site model and delivery	Contaminant distribution, depth, moisture, permeability, active zone, delivery route, and monitoring access	Evidence that the treatment reached the limiting zone	Treatment added but not shown to contact the active fraction
Endpoint and land-use link	Predefined endpoint tied to land use, exposure pathway, or regulatory decision	Endpoint met without changing the endpoint after results are known	Endpoint undefined, revised *post hoc*, or unrelated to exposure
Organic transformation safety	Parent compounds, expected products, pathway conditions, and toxicity plan	Parent decline with product control and toxicity reduction	Persistent hazardous intermediates or increased toxicity
Metal and metalloid risk	Speciation, pore-water fraction, leachability, pH, Eh, and plant-uptake risk	Stable reduction in mobility, bioaccessibility, or exposure under relevant site changes	Remobilization under pH, redox, wetting-drying, or root-zone conditions
Biological attribution	Activity markers selected before treatment	Activity-linked evidence converges with contaminant and toxicity endpoints	Organisms, genes, or transcripts present without endpoint change
Bioaugmentation and SynCom control	Product identity, carrier, dose, delivery, member tracking, and safety plan	Function persists after dosing or active support stops	Persistence without function, loss of key member, or rebound
Nano-enabled treatment control	Material identity, dose, particle fate, dissolution, aggregation, mobility, and toxicity plan	Contaminant benefit without unacceptable particle movement or biological impairment	Apparent removal with particle mobility, toxicity, or unmeasured fate
AI-supported decision	Decision claim, input variables, validation design, uncertainty, and applicability domain	Model performance remains valid for the site decision and does not replace endpoint evidence	Calibration-only accuracy, domain shift, or decision not linked to field action
Durability	Monitoring window matched to failure mode	No rebound, remobilization, loss of activity, or renewed exposure after active treatment stops	Rebound, remobilization, or function loss

AI, artificial intelligence; Eh, redox potential; SynCom, synthetic microbial community.

A final implication follows for reporting. Field-ready studies should state not only what improved, but what evidence would invalidate the conclusion. That counter-evidence may be rebound, toxic intermediate persistence, nanoparticle mobility, loss of microbial function, biomass exposure, model failure outside its domain, or inability to deliver the treatment. A closure claim without predefined failure criteria is weaker than its apparent data volume suggests. Emerging contaminant classes strengthen this reporting requirement because they expose the weakness of closure claims based on concentration decline alone. PFAS, microplastics, and complex contaminant mixtures require explicit separation among biological transformation, abiotic transformation, immobilization, extraction, exposure interruption, and verified destruction. For PFAS in particular, lower leaching or pore-water concentration after sorptive amendment should not be interpreted as destruction unless fluorine mass balance, precursor behavior, transformation products, and residual hazard are addressed (Liang et al., 2024). Recent syntheses of soil-pollution challenges likewise show that metals, pesticides, PFAS, microplastics, sensors, and AI-supported monitoring require closure frameworks that specify what has changed, by which mechanism, and with what remaining exposure pathway (Chen H. et al., 2025).

## 9 Conclusion and future directions

Soil bioremediation fails most often when the evidence claim is stronger than the process demonstrated in soil. Parent-compound loss is not necessarily detoxification. Immobilization is not destruction. Inoculant survival is not function. Plant growth is not phytoremediation success unless the endpoint is defined. Omics, isotope tools, SynComs, nanomaterials, AI, and digital twins can improve decisions only when they reduce uncertainty around a diagnosed failure mode.

The main contribution of this review is a field-facing decision sequence: diagnose the failure mode, identify the limiting mechanism, select the least complex admissible intervention, test pathway operation and secondary risk, and close only when endpoint durability matches intended land use. This sequence is intended to prevent premature escalation from poor performance to more complex technology.

Future work should concentrate on five priorities. First, field and pilot studies should report negative, partial, and conditional outcomes with the same care as successful outcomes. Second, contaminant-product chemistry, isotope evidence, activity-linked molecular monitoring, toxicity assays, and post-treatment monitoring should be combined explicitly when biological attribution or closure is claimed. Third, nano-enabled systems, SynComs, and engineered microorganisms should be tested as bounded interventions with fate, function, containment, and secondary-risk evidence. Fourth, AI models and digital twins should report the decision claim, applicability domain, uncertainty, monitoring triggers, and update rules before they are used to guide field action. Fifth, closure should include ecological recovery where soil function is part of the remediation objective, because reduced contaminant pressure alone does not prove microbiome reassembly or functional resilience. Under this standard, soil bioremediation remains credible when mechanism, evidence, intervention, and closure are kept aligned.
